# *CircUCK2(2,3)* promotes cancer progression and enhances synergistic cytotoxicity of lenvatinib with EGFR inhibitors via activating CNIH4–TGFα–EGFR signaling

**DOI:** 10.1186/s11658-025-00690-1

**Published:** 2025-01-30

**Authors:** Xindong Wei, Anfeng Si, Shuai Zhao, Yi Fu, Jilei Li, Kedeerya Aishanjiang, Yujie Ma, Chang Yu, Bo Yu, Chunhong Cui, Hui Wang, Xianming Kong, Shibo Li, Xiaoni Kong, Ying Tong, Hailong Wu

**Affiliations:** 1 Clinical Research Center, Jiading District Central Hospital Affiliated to Shanghai University of Medicine and Health Sciences, Shanghai, 201800 China; 2 Central Laboratory, ShuGuang Hospital Affiliated to Shanghai University of Chinese Traditional Medicine, Shanghai, 201203 China; 3 Collaborative Research Center for Biomedicines, Shanghai University of Medicine and Health Sciences, Shanghai, 201318 China; 4 Department of General Surgery, Jinling Hospital, Affiliated Hospital of Medical School, Nanjing University, Nanjing, 210015 China; 5 Department of Transplantation, Xinhua Hospital Affiliated to Shanghai Jiao Tong University School of Medicine, Shanghai, 200092 China; 6 People’s Hospital of Xinjiang Uygur Autonomous Region, Urumqi, 831399 China; 7 School of Clinical Medicine, Shanghai University of Medicine and Health Sciences, Shanghai, 201318 China; 8 Basic Medical College, Shanghai University of Medicine and Health Sciences, Shanghai, 201318 China; 9 Department of Infectious Disease, Zhoushan Hospital, Wenzhou Medical University, Zhoushan, 316100 China; 10 Department of Liver Surgery, School of Medicine, Renji Hospital, Shanghai JiaoTong University, Shanghai, 200003 China; 11 School of Pharmacy, Joint Innovation Laboratory for Cell Therapy Technology, Shanghai University of Medicine and Health Sciences, Shanghai, 201318 China

**Keywords:** Hepatocellular carcinoma, circRNA, CNIH4, TGFα–EGFR signaling, Lenvatinib, EGFR inhibitors

## Abstract

**Background:**

Circular (circ)RNAs have emerged as crucial contributors to cancer progression. Nonetheless, the expression regulation, biological functions, and underlying mechanisms of circRNAs in mediating hepatocellular carcinoma (HCC) progression remain insufficiently elucidated.

**Methods:**

We identified *circUCK2(2,3)* through circRNA sequencing, RT–PCR, and Sanger sequencing. *CircUCK2(2,3)* levels were measured in two independent HCC cohorts using quantitative real-time PCR (qRT–PCR). We explored the functions of *circUCK2(2,3)* using gain- and loss-of-function assays. Techniques such as RNA-sequencing, RNA immunoprecipitation (RIP), polysome fractionation, RNA pulldown, dual luciferase reporter assay, inhibitors of EGFR downstream signaling, CRISPR–Cas9, and medium transfer assays were employed to investigate the regulatory mechanisms and the protumoral activities of *circUCK2(2,3)*. Additionally, in vitro cytotoxic assays and patient-derived xenograft (PDX) models assessed the effects of *circUCK2(2,3)* on the cytotoxic synergy of lenvatinib and EGFR inhibitors.

**Results:**

*CircUCK2(2,3)* is upregulated in HCC tissues and serves as an independent risk factor for poor recurrence-free survival. The expression of *circUCK2(2,3)* is independent on its host gene, *UCK2*, but is regulated by its upstream promoter and flanking inverted complementary sequences. Functionally, *circUCK2(2,3)* enhances HCC proliferation, migration, and invasion, both in vitro and in vivo*.* Mechanistically, by sponging miR-149-5p, *circUCK2(2,3)* increases CNIH4 levels, which in turn amplifies TGFα secretion, resulting in the activation of EGFR and downstream pAKT and pERK signaling pathways. Moreover, *circUCK2(2,3)* overexpression sensitizes HCC cells to EGFR inhibitors, and increases the synergistic cytotoxicity of combined lenvatinib and EGFR inhibitor treatment.

**Conclusions:**

*CircUCK2(2,3)* regulates a novel oncogenic pathway, miR-149-5p–CNIH4–TGFα–EGFR, in HCC, presenting a viable therapeutic target and biomarker for the precision treatment of HCC.

**Graphical Abstract:**

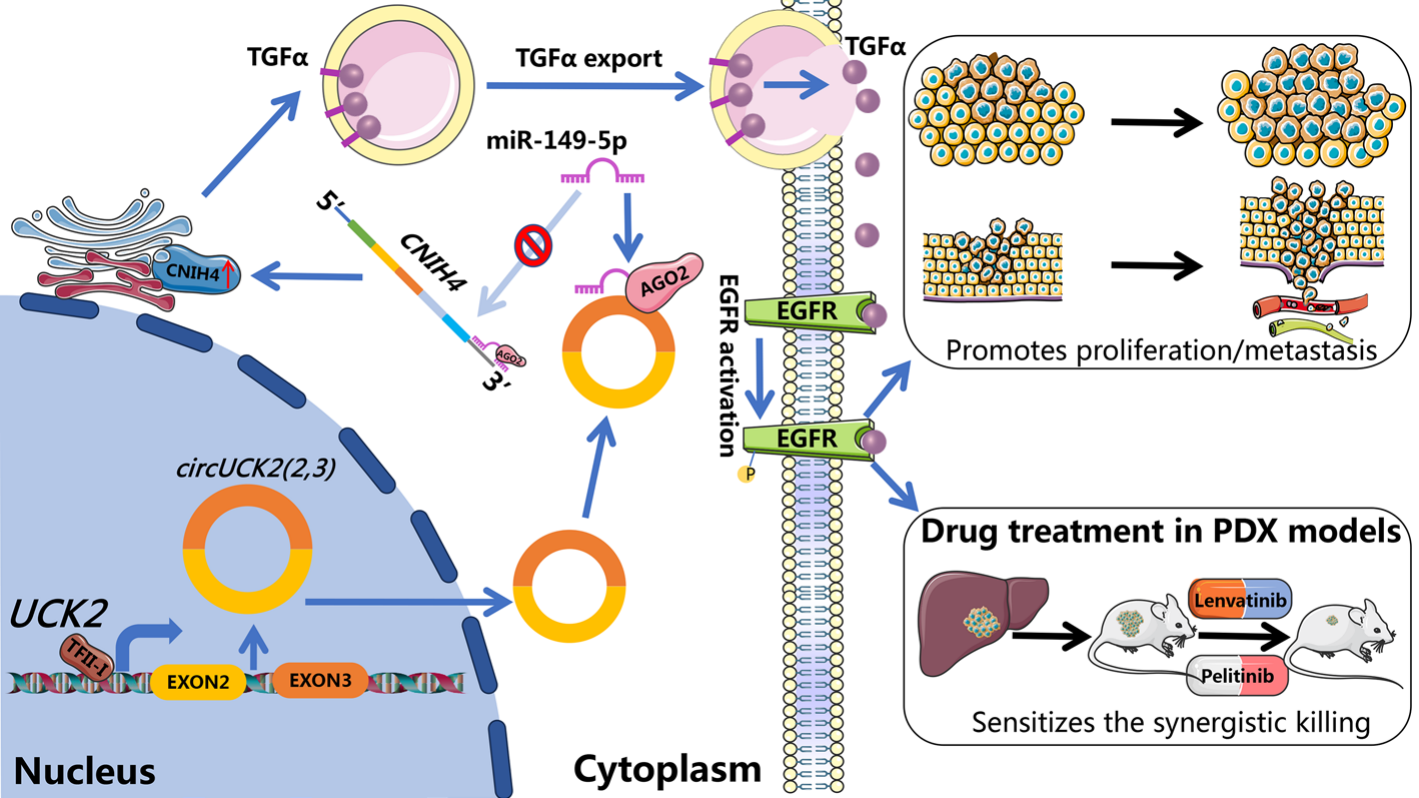

**Supplementary Information:**

The online version contains supplementary material available at 10.1186/s11658-025-00690-1.

## Background

Hepatocellular carcinoma (HCC) is the most common form of primary liver cancer and a leading cause of cancer-related mortality worldwide [1]. Unfortunately, HCC is often diagnosed at advanced stages, with poor prognoses due to asymptomatic early stages [2]. Despite advancements in systemic therapies such as tyrosine kinase inhibitors (TKIs) and immune checkpoint inhibitors (ICIs) [3], the prognosis for HCC patients remains unsatisfactory, largely attributed to drug resistance [4]. Therefore, there is an urgent need to identify novel therapeutic targets for HCC treatment.

Circular RNAs (circRNAs) are a unique subgroup of noncoding RNAs characterized by their covalently closed circular structure [5]. This circular structure confers circRNAs with increased stability and resistance to degradation compared to linear transcripts. Accumulating evidence suggests that circRNAs play significant roles in various pathological processes, including cancer [6–9]. Previous studies have reported oncogenic or tumor-suppressive functions of circRNAs in several cancer types, including lung cancer [10], breast cancer [11], nasopharyngeal carcinoma [12], leukemia [13], oral squamous cell carcinoma [7], bladder cancer [14], and glioblastoma [15]. In HCC, specific circRNAs have been identified, such as exosomal *circCCAR1* promoting anti-PD-1 resistance [16], and *circASH2* suppressing HCC metastasis through the regulation of *TPM4* expression [17]. However, our understanding of the roles and mechanisms of circRNAs in HCC progression remains limited.

TGFα functions as a potent mitogen of normal and neoplastic hepatocytes. TGFα overexpression and secretion has been closely associated with HCC initiation and progression [18]. TGFα-induced HCC formation is mainly attributed to the oncogenic activation of the EGFR pathway [19]. *CNIH4* belongs to the evolutionarily conserved CORNICHON family, which is involved in the intracellular transport and export of TGFα [20–22]. According to the Catalogue Of Somatic Mutations In Cancer (COSMIC) database [23], *CNIH4* overexpression has been detected in multiple cancer types including liver cancer. In HCC, copy number variation (CNV) gain and overexpression of *CNIH4* have been observed in a subset of cases, suggesting its potential involvement in HCC progression. The protumor function of *CNIH4* has been reported in glioblastoma, head and neck squamous cell carcinoma, gastric cancer, and colon cancer [24–28]. Additionally, *CNIH4* is part of a five-gene signature predicting the overall survival of HCC patients [29]. However, the specific role of *CNIH4* in HCC remains unexplored.

In this study, we identified *circUCK2(2,3)* (circBase_ID: hsa_circ_0006758) as being significantly upregulated in HCC tissues across two independent HCC cohorts. The expression of *circUCK2(2,3)* is correlated with poorer tumor differentiation and serves as an independent risk factor for shorter recurrence-free survival in HCC patients. *CircUCK2(2,3)* expression is regulated by an upstream 90 nt element, the transcriptional factor TFII-I, and the flanking *Alu* elements. Our in vitro and in vivo models demonstrate that *circUCK2(2,3)* enhances HCC cell proliferation, migration, and invasion, and increases the sensitivity of HCC cells to the cytotoxicity of EGFR inhibitors, as well as the synergistic cytotoxic effect of lenvatinib combined with EGFR inhibitors. This effect is attributed to *circUCK2(2,3)*-mediated alleviation of miR-149-5p inhibition on *CNIH4*, leading to increased TGFα secretion and subsequent EGFR activation. Therefore, we have identified a novel regulatory axis, *circUCK2(2,3)*–miR-149-5p–CNIH4–TGFα–EGFR, which contributes to HCC development.

## Methods

### Clinical HCC tissue samples

Studies using human tissues were approved by the Institutional Ethical Review Boards of Jinling Hospital, Medical School of Nanjing University (DZQH-KYLL-24-01) and Xinhua Hospital Affiliated to Shanghai Jiao Tong University School of Medicine (XHEC-D-2023-109), and performed in accordance with the principles of Declaration of Helsinki. Forty paired HCC tissues and peritumor tissues were collected at the Department of Transplantation Surgery, Xinhua Hospital, Shanghai Jiao Tong University and termed as the Xin-Hua cohort. Seventy paired HCC tissues and peritumor tissues were collected at the Department of Surgical Oncology, Jinling Hospital, Medical School of Nanjing University and termed as the Jin-Ling cohort. None of the patients had received preoperative anticancer treatments. Written informed consent was obtained from all patients to use clinical information. All of the fresh tissues were snap-frozen in liquid nitrogen and then stored at −80 °C for further study.

### CircRNA sequencing and analysis

Five paired HCC tissues and peritumor tissues were selected for circRNA sequencing (Majorbio, Shanghai, China). Briefly, total RNA was isolated using TRIzol reagent. The Ribo Zero Magnetic kit (Epicentre Biotechnologies) was used to remove ribosomal RNA from samples. Noncircular RNAs were removed by RNase R treatment, and fragmentation of circRNAs was performed with magnesium-based RNA cleavage. Subsequently, the TruSeq Stranded Total RNA Library Prep kit (Illumina Inc., CA, USA) was used to prepare RNA-sequencing (RNA-seq) libraries, which were then sent to Majorbio for deep sequencing on the Illumina HiSeq4000 system (Illumina Inc., CA, USA). The FASTQ reads were aligned to the human reference genome (hg38/GRCh38). CIRI2 was used to identify circRNA. Briefly, during the first scanning of SAM alignment, CIRI2 detects junction reads with paired chiastic clipping (PCC) signals that reflect a circRNA candidate. Preliminary filtering was implemented using paired-end mapping (PEM) and GT–AG splicing signals for the junctions. After clustering junction reads and recording each circRNA candidate, CIRI scanned the SAM alignment again to detect additional junction reads and meanwhile performed further filtering to eliminate false positive candidates resulting from incorrectly mapped reads of homologous genes or repetitive sequences. To identify differentially expressed (DE)circRNAs between HCC tissues and peritumor tissues, the expression level of each circRNA was calculated according to the reads per million mapped reads (RPM) method. CircRNAs whose expression was detectable in all five HCC tissues were selected for differential expression analysis using DEGseq with |log2 (fold change)|≥ 1 and *p*-adjust ≤ 0.05 considered to be significantly differentially expressed circRNAs.

### RNA Immunoprecipitation assay (RIP)

The AGO2 RIP assay was performed by using the Magana RIP RNA-Binding Protein Immunoprecipitation kit (Millipore, USA) according to the manufacturer’s protocol. Briefly, 2 × 10^7^ cells were lysed in 200 μl lysis buffer containing a protease inhibitor cocktail and RNase inhibitor. Then, 0.5% of the cell lysate (1 ml) was transferred to a clean tube as input. Magnetic beads were pre-incubated with the anti-AGO2 antibody (CST #2897) or isotype control (CST #3900) for 1 h at room temperature, and then incubated with cell lysates at 4 °C overnight. The RNA precipitates were purified with the miRNeasy Mini kit (Qiagen, USA) and subjected to quantitative real-time PCR (qRT–PCR) analysis. Information on the antibodies used in this study is listed in Supplementary Table 5.

### Nucleus/cytoplasm fractionation

The separation of nuclear and cytoplasmic fractions from cultured cells was conducted using the PARIS kit (Thermo Scientific, AM1556) following the manufacturer’s instructions. The separation efficiency was determined by measuring the nuclear or cytoplasmic distribution of *MALAT1* [a well-known nuclear long noncoding RNA (lncRNA), a type of noncoding transcripts of more than 200 nucleotides], and *BIRC5* (a well-known cytoplasmic lncRNA). Briefly, PLC/PRF/5 and SNU182 cells were harvested and resuspended in Cell Fraction Buffer on ice for 10 min. The lysates were centrifuged at 500 g for 5 min at 4 °C. The supernatant was collected and saved as the cytoplasmic extracts. After a thorough wash with the Cell Fraction Buffer, the pellet was saved as the nuclear extract.

### In vivo tumor growth and metastasis assays

The procedures for animal experiments were approved by the Institutional Animal Care and Use Committee of Shanghai University of Medicine and Health Sciences (SUMHS-IACUC-2022-0221). Male athymic BALB/c nude mice (4–5 weeks old) were purchased from the Vital River Laboratories (Shanghai, China), and housed in individual micro-isolator cages under standard pathogen-free conditions of 22 ± 2 ℃ and 55 ± 5% humidity, with free access to food and water. For the subcutaneous xenograft study, BALB/c nude mice were randomly divided into six groups (*n*= 5–6 per group). PLC/PRF/5 cells (5 × 10^6^) with or without *circUCK2(2,3)* knockdown, as well as SNU398 cells (5 × 10^6^) with wild-type (WT) or 149-mut *circUCK2(2,3)* overexpression were subcutaneously implanted into the right flank of the nude mice. Tumor sizes were measured with a caliper three times weekly from the eighth day after injection. Tumor volume was calculated using the following equation: tumor volume (mm^3^) = shorter diameter^2^ (mm^2^) × longer diameter (mm)/2. All the mice were sacrificed at the indicated time and the tumors were removed for further studies. For lung metastasis study, BALB/c nude mice were randomly divided into six groups (*n* = 5 per group). PLC/PRF/5 cells with stable *circUCK2(2,3)* knockdown (2 × 10^6^ in 100 μl PBS), or SNU398 stable transfectants overexpressing WT *circUCK2(2,3)* or 149-mu *circUCK2(2,3)* (2 × 10^6^ in 100 μl PBS) were injected into nude mice via tail vein. At 8 weeks after injection, mice were sacrificed and the lung tissues were fixed in Bouin’s solution (Sigma-Aldrich, HT10132) and embedded in paraffin for histological hematoxylin and eosin staining.

### *CircUCK2(2,3)* pulldown experiment

The *CircUCK2(2,3)* pulldown experiment was performed by a chromatin isolation by RNA purification (ChIRP) assay [30]. Briefly, HCC cells were first crosslinked with 4% glutaraldehyde for 10 min and the crosslinking reaction was quenched with 1/10 volume of 1.25 M glycine at room temperature for 5 min, and supplemented with 1 ml cell lysis buffer [50 mM Tris–Cl pH 7.0, 10 mM EDTA, 1% SDS, fresh protease inhibitor, phenylmethylsulfonyl fluoride (PMSF), and RNase inhibitor] per 100 mg of cell pellet. After cell lysis and sonication, 1% of cell lysate (10 μl) was removed for RNA input. Biotin-labeled DNA probes targeting *circUCK2(2,3)* (circ-probes) or linear *UCK2* (lin-probes) (Ribobio Co, Shanghai, China) were added and incubated with cell lysate at 37 °C for 4 h. The sequence information of probes is provided in Supplementary Table 4. Streptavidin-conjugated Dynabead M-280 beads (Thermo Scientific, M-280) were then added into the mixture of cell lysate and probes,followed by a 30 min incubation at 37 °C. After incubation, half of the Dynabeads were collected, cleaned, and digested with proteinase K to remove the protein for subsequent qRT–PCR assays to detect the enrichment of *circUCK2(2,3)* or TaqMan miRNA assays to detect the binding miRNAs of *circUCK2(2,3)*. The other half of the Dynabeads was boiled in loading buffer at 95–100 °C for 5 min for subsequent western blotting to detect the binding between *circUCK2(2,3)* and AGO2.

### Medium transfer assay

Stable SNU398 transfectants with WT *circUCK2(2,3)* or 149-mut *circUCK2(2,3)* overexpression were seeded into a 6-well plate at 5 × 10^5^/well. After 24 h of culture, the conditional medium was filtered through a 0.2 μm membrane filter to avoid contamination of the donor cells, and then transferred to starved serum (24 h pre-culture in serum-free medium). After 24 h incubation with conditional medium, cell lysates of SNU398 were collected by RIPA lysis buffer supplied with protease inhibitors and phosphatase inhibitors. Phosphorylation changes of EGFR were detected by western blotting.

### Synergy matrices

SNU398 cells with or without *circUCK2(2,3)* overexpression were seeded at 96-well plates at 5000 cells/well. After overnight incubation, culture medium with indicated drug combinations was added. The cytotoxicity of the combined drugs was determined by counting kit-8 (CCK8) assays at 48 h after drug treatment. The readings were normalized using the mean of negative controls (DMSO, 0% inhibition) to obtain relative inhibition percentage, and then uploaded on https://synergyfinder.org to calculate the respective synergy scores (Bliss). Three independent experiments were performed to calculate the average synergy scores (Bliss) at indicated drug combinations. Drug information is listed in Supplementary Table 5.

### Long-term synergistic killing effects

Clonogenic assays were employed to determine the long-term synergistic killing effects of lenvatinib combined with EGFR inhibitors. Briefly, SNU398 cells were plated in replicate 12-well plates at 10,000 cells/well and incubated overnight to allow for attachment. The indicated drug combinations were then added to the cells. The culture media, with or without drugs, were refreshed every 3 days for up to 2 weeks. When the cell density in the control wells reached approximately 90% confluence, cells were fixed and stained with 0.5% crystal violet in 4% paraformaldehyde for 30 min. All viability measurements were expressed as a percentage of the DMSO-treated control.

### Patient-derived xenografts (PDX) establishment

The PDX protocol received approval from the Institutional Review Board at Xinhua Hospital, affiliated with the Shanghai Jiao Tong University School of Medicine. Briefly, HCC samples were obtained and cut into 3 mm^3^ sections, and then implanted into the subcutaneous tissue of the anterior flanks of female BALB/c nude mice, aged 6–8 weeks, under anesthesia within a 2 h timeframe. Once the tumors reached approximately 200 mm^3^ in volume, the mice were randomly assigned to receive treatment 5 days a week with either a vehicle control, lenvatinib (administered at a dose of 4 mg/kg via oral gavage), pelitinib (administered at a dose of 10 mg/kg via oral gavage), or a combination of both drugs. The individual drugs in the combination were given at the same dosage and on the same schedule as when they were used as single agents. Tumor volume was determined using caliper measurements and calculated with the modified ellipsoidal formula: tumor volume = 1/2 × length × (width)^2^. For the survival curve analysis, treatments were continued until the tumors reached a total volume of 2000 mm^3^. All procedures and protocols were approved by the Institutional Animal Care and Use Committee at Shanghai University of Medicine and Health Sciences.

### Statistical analysis

Quantitative data were compared between groups using Student’s *t*-test. Categorical data were analyzed by the chi-squared test or Fisher’s exact test. Multivariate analyses were performed by multivariate Cox proportional hazard regression model. Correlation analyses were performed between *circUCK2(2,3)*, *UCK2*, and *CNIH4*. Overall survival and cumulative recurrence rates were calculated by the Kaplan–Meier method, and differences were analyzed by the log-rank test. Tumor growth curves were analyzed by two-way ANOVA. *p* < 0.05 was considered statistically significant.

## Results

### Identification and characterization of *circUCK2(2,3)* in HCCs

**Fig. 1:**
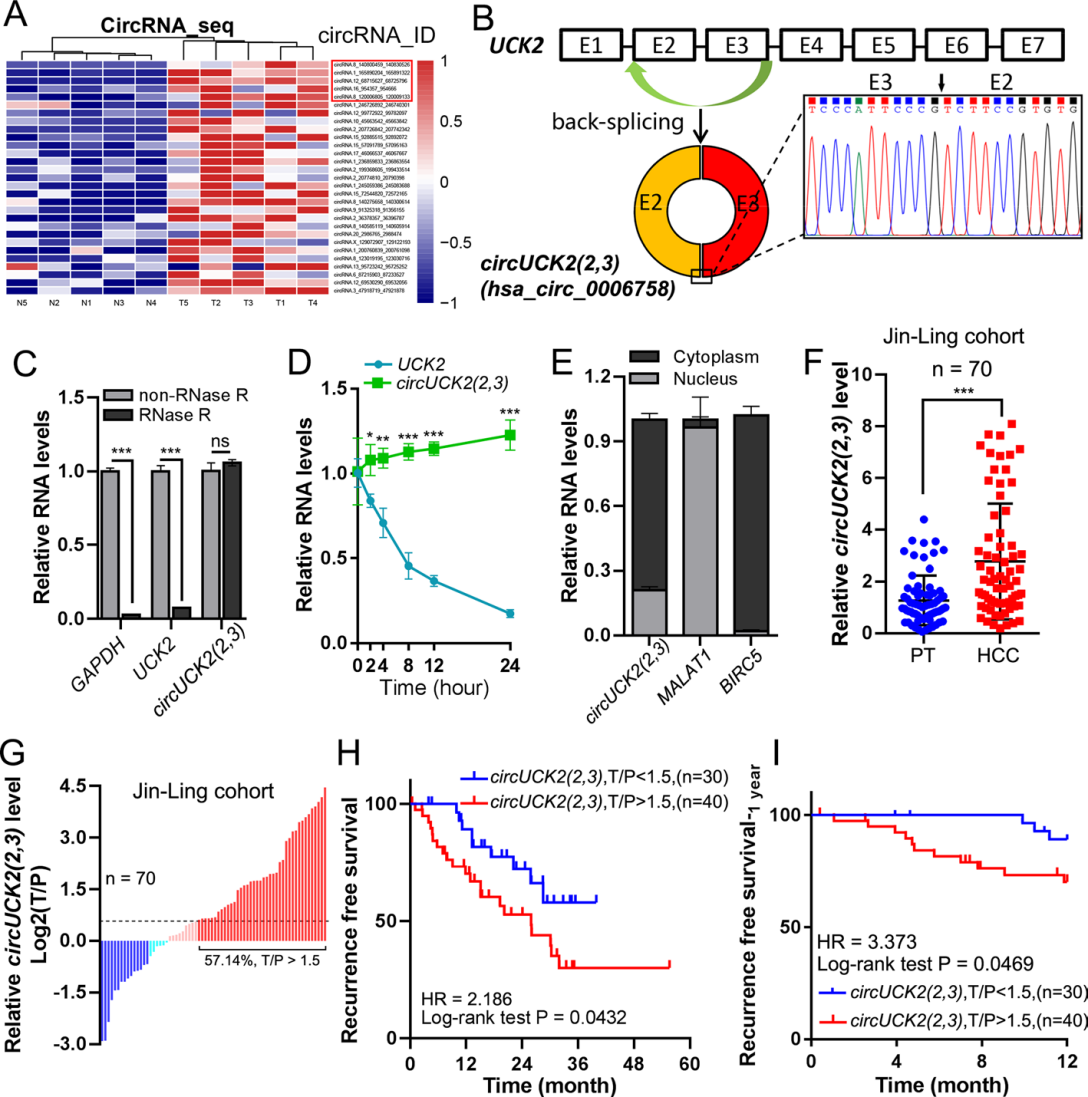
Identification and characteristics of *circUCK2(2,3)* in HCC. **A** Heat map of upregulated circRNAs in five HCC tissues compared to matched normal tissues (the top five upregulated circRNAs are highlighted with a red rectangle box). **B** Schematic showing the generation of *circUCK2(2,3)* from exon 2 and exon 3 of *UCK2*. The nucleotide sequence of the back-splicing site of *circUCK2(2,3)* was confirmed by Sanger sequencing. **C** and **D** qRT–PCR assessed the resistance of *circUCK2(2,3)* to RNase R treatment (**C**), and evaluated the stability of *circUCK2(2,3)* after actinomycin D treatment (**D**). **E** qRT–PCR assessment of the nuclear or cytoplasmic distribution of *circUCK2(2,3)*. **F** Relative expression of *circUCK2(2,3)* in the Jin-Ling cohort, containing 70 paired HCC and peritumor tissues. **G** Tumor versus peritumor expression ratio of *circUCK2(2,3)* in clinical samples of the Jin-Ling cohort. **H** and **I** Kaplan–Meier curve showing the association between *circUCK2(2,3)* expression and recurrence free survival at 5 years (**H**) or 1 years (**I**) post surgery. Survival curves were compared using the log-rank test. Data are shown as the mean ± SD. Statistical analyses were performed using paired Student’s *t*-tests (NS, no statistical significance; *p < 0.05; **p < 0.01; ***p < 0.001). *UCK2* uridine-cytidine kinase 2, *circUCK2(2,3)* circRNA uridine-cytidine kinase 2, *GAPDH* glyceraldehyde-3-phosphate dehydrogenase, *MALAT1* metastasis associated lung adenocarcinoma transcript 1, *BIRC5* baculoviral IAP repeat containing 5, *HCC* hepatocellular carcinoma, *PT* peritumor, *T/P* tumor versus peritumor

To identify circRNAs associated with HCC progression, we performed circRNA sequencing (circRNA-seq) on five paired HCC tissues and adjacent peritumor tissues. CircRNA-seq results revealed that there were 29 circRNAs significantly upregulated (*p* < 0.05, fold change > 2) in HCC tissues (Fig. 1A). Based on their *p*-values, we selected the top five upregulated circRNAs for further validation (Fig. 1A, red border rectangle). Reverse transcription PCR (RT-PCR) in HCC tumor tissue (T4), followed by Sanger sequencing, confirmed the expression of two out of five circRNA candidates (Fig. S1A). qRT–PCR identified that candidate 2 (circRNA.1_165890204-165891322) was the most upregulated (Fig. S1B), leading to its selection for further study. The official name of this circRNA in the circBase database (http://circbase.org/) is hsa_circ_0006758, which spans 257 nucleotides and is derived from back-spliced exons 2 and 3 of the *UCK2* gene (Fig. 1B). Following the new naming guidance of eukaryotic circRNAs [31], we therefore named hsa_circ_0006758 as “*circUCK2(2,3)*,” indicating its origin from exons 2 and 3 of the *UCK2* gene.

Unlike linear transcripts whose cDNA can be synthesized using either oligo (dT) or random primers, the cDNA of *circUCK2(2,3)* can only be generated using random primers (Fig. S1C). Compared to its linear counterpart, *circUCK2(2,3)* displayed resistance to RNase R digestion (Fig. 1C and Fig. S1D) and actinomycin D treatment (Fig. 1D). Next, qRT–PCR after nucleus/cytoplasm fractionation (Fig. 1E), and RNAscope in situ hybridization (Fig. S1E), determined the cytoplasmic distribution of endogenous *circUCK2(2,3)* in PLC/PRF/5 cells.

To assess the pathological relevance of *circUCK2(2,3)*, we evaluated its expression in two clinical HCC cohorts, the Jin-Ling and the Xin-Hua cohorts. Compared to peritumor tissues, *circUCK2(2,3)* was significantly upregulated in HCC tissues (Fig. 1F, Fig. S1F–H). Over 57% of HCC samples demonstrated marked upregulation of *circUCK2(2,3)* (fold change > 1.5) in both cohorts (Fig. 1G and Fig. S1I). The expression level of *circUCK2(2,3)* was associated with multiple clinicopathological characteristics, including patient age, presence of HBsAg, and poor tumor differentiation (Supplementary Table S1). Multivariate Cox regression further demonstrated that *circUCK2(2,3)* is an independent prognostic predictor for recurrence-free survival (RFS) (Supplementary Table S2). Moreover, Kaplan–Meier analyses showed that *circUCK2(2,3)* negatively correlates with RFS and early-RFS (1 year post-surgical resection) in HCC patients (Fig. 1H, I). Consistent with previous findings showing elevated *UCK2* expression in HCC tissues [32], more than 85% and 77% of HCC samples in the Jin-Ling and the Xin-Hua cohorts showed marked *UCK2* upregulation, respectively (fold change > 1.5) (Fig. S1J and K). However, a weak expression correlation was observed between *circUCK2(2,3)* and linear *UCK2* in both HCC cohorts (Fig. S1L and M), suggesting that the formation of *circUCK2(2,3)* and *UCK2* mRNA may not in a competitive model. Therefore, these findings indicate that *circUCK2(2,3)* is upregulated in HCC tissues and is associated with poor prognosis.

### The protumor effects of *circUCK2(2,3)*

To evaluate the function of *circUCK2(2,3)*, we first examined its expression in HCC cell lines. *CircUCK2(2,3)* expression was high in Hep3B, SNU182, and PLC/PRF/5 cell lines, and low in SNU398, SK-HEP-1, and MHCC-97H cell lines (Fig. S2A). To investigate its role, we knocked down *circUCK2(2,3)* in SNU182 and PLC/PRF/5 cells, and overexpressed it in SNU398 and SK-HEP-1 cells (Fig. S2B and C). Neither knockdown nor overexpression of *circUCK2(2,3)* affected *UCK2* expression (Fig. S2D and E), indicating no transcriptional regulatory interactions between *circUCK2(2,3)* and *UCK2*. Meanwhile, RNAscope in situ hybridization showed that ectopic *circUCK2(2,3)* overexpression in SK-HEP-1 cells did not change its cytoplasmic distribution (Fig. S2F).

**Fig. 2:**
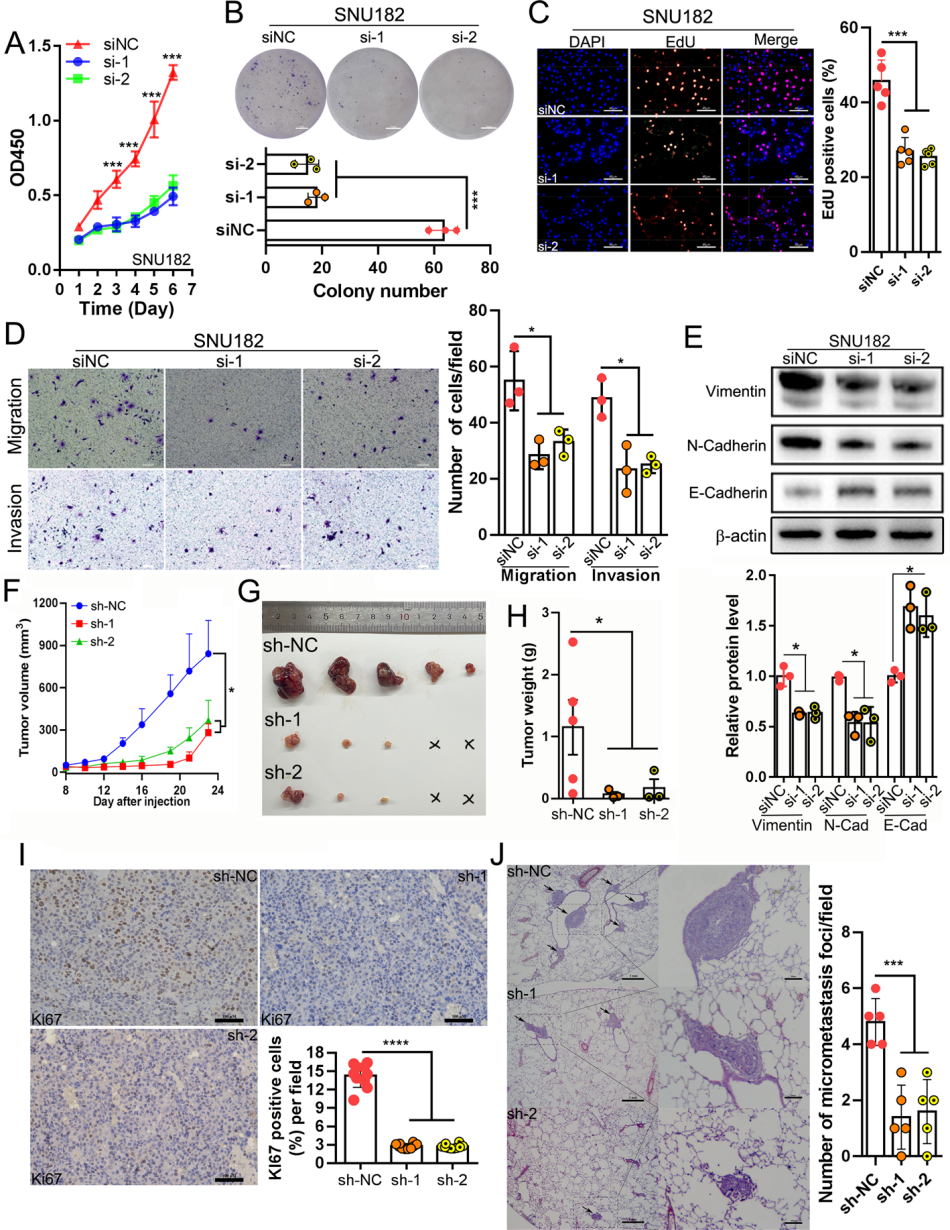
*CircUCK2(2,3)* promotes HCC cell proliferation and metastasis both in vitro and in vivo. **A** and **B** CCK8 assays (**A**) or colony formation assays (**B**) in SNU182 cells with *circUCK2(2,3)* knockdown, (scale bar, 5 mm). **C** EdU staining assays in SNU182 cells with *circUCK2(2,3)* knockdown, (scale bar, 40 μm). **D** Transwell migration and invasion assays in SNU182 cells with *circUCK2(2,3)* knockdown, (scale bar, 40 μm). **E** Western blotting and densitometry of EMT markers in SNU182 cells with *circUCK2(2,3)* knockdown. **F**–**H** PLC/PRF/5 cells with stable *circUCK2(2,3)* knockdown were injected into nude mice subcutaneously. Seven days after injection, tumor volumes were measured three times weekly by using a caliper to draw tumor growth curve (**F**) (means ± SD, two-way ANOVA). After 23 days of injection, subcutaneous tumors were dissected and imaged (**G**). Tumor weight was compared between the sh-NC group and the groups with *circUCK2(2,3)* knockdown (**H**). **I** IHC staining of Ki67 in xenograft tumors (scale bar, 100 μm). **J** PLC/PRF/5 cells with stable *circUCK2(2,3)* knockdown were introduced into nude mice intravenously. Eight weeks after the initial injection, lungs were dissected and lung micrometastases were detected by H&E staining (scale bar, 1 mm and 200 μm). Statistical analyses were performed using paired Student’s *t*-tests (*p < 0.05; **p < 0.01; ***p < 0.001; ****p < 0.0001). *siNC* negative control of siRNA, *si-1* siRNA#1, *si-2* siRNA#2, *VC* vector control, *OE* overexpression, *DAPI* 4′,6-diamidino-2-phenylindole, *EdU* 5-ethynyl-2′-deoxyuridine, *sh-NC* negative control of shRNA, *sh-1* shRNA#1, *sh-2* shRNA#2, *Ki67* marker of proliferation

CCK-8 and long-term colony formation assays revealed that *circUCK2(2,3)* knockdown reduced, while overexpression enhanced, HCC cell proliferation (Fig. 2A, B, Fig. S3A–D). Correspondingly, EdU staining and EdU flow cytometry showed decreased or increased S-phase populations in HCC cells upon *circUCK2(2,3)* knockdown or overexpression, respectively (Fig. 2C, Fig. S3E–J). Additionally, wound healing, transwell migration, and invasion assays demonstrated that *circUCK2(2,3)* knockdown impaired, while overexpression enhanced, HCC cell migration and invasion (Fig. 2D, Fig. S4A–E). Western blot further confirmed that *circUCK2(2,3)* knockdown decreased EMT markers of vimentin and N-cadherin, and increased E-cadherin in SNU182 and PLC/PCR/5 cells (Fig. 2E, and Fig. S4F), whereas *circUCK2(2,3)* overexpression had the opposite effect in SNU398 and SK-HEP-1 cells (Fig. S4F).

Moreover, a subcutaneous xenograft nude mouse model demonstrated that stable *circUCK2(2,3)* knockdown significantly reduced tumor growth, size, and weight in PLC/PRF/5 cells in vivo (Fig. 2F–H). Ki67 IHC staining indicated that *circUCK2(2,3)* knockdown severely impaired HCC cell proliferation in vivo (Fig. 2I). A lung metastasis model in nude mice showed that constant *circUCK2(2,3)* knockdown in PLC/PRF/5 cells significantly decreased the number and size of lung micrometastases (Fig. 2J). These findings suggest that *circUCK2(2,3)* functions as an oncogenic circRNA in HCC progression.

### *CircUCK2(2,3)* functions as a sponge of miR-149-5p

*CircRNAs* execute their functions by serving as miRNA sponges, protein scaffolds, or translatable templates. Using the CPAT (http://lilab.research.bcm.edu/) and CPC2 (http://cpc2.gao-lab.org/) databases [33, 34], we found no protein-coding potential of *circUCK2(2,3)* (Fig. S5A). Additionally, a polysome fractionation analysis confirmed the noncoding nature of *circUCK2(2,3)* (Fig. S5B). The CircInteractome database indicated that *circUCK2(2,3)* has six potential AGO2 binding sites (Fig. S5C and D) [35], suggesting that *circUCK2(2,3)* may function as a miRNA sponge in promoting HCC progression.

**Fig. 3:**
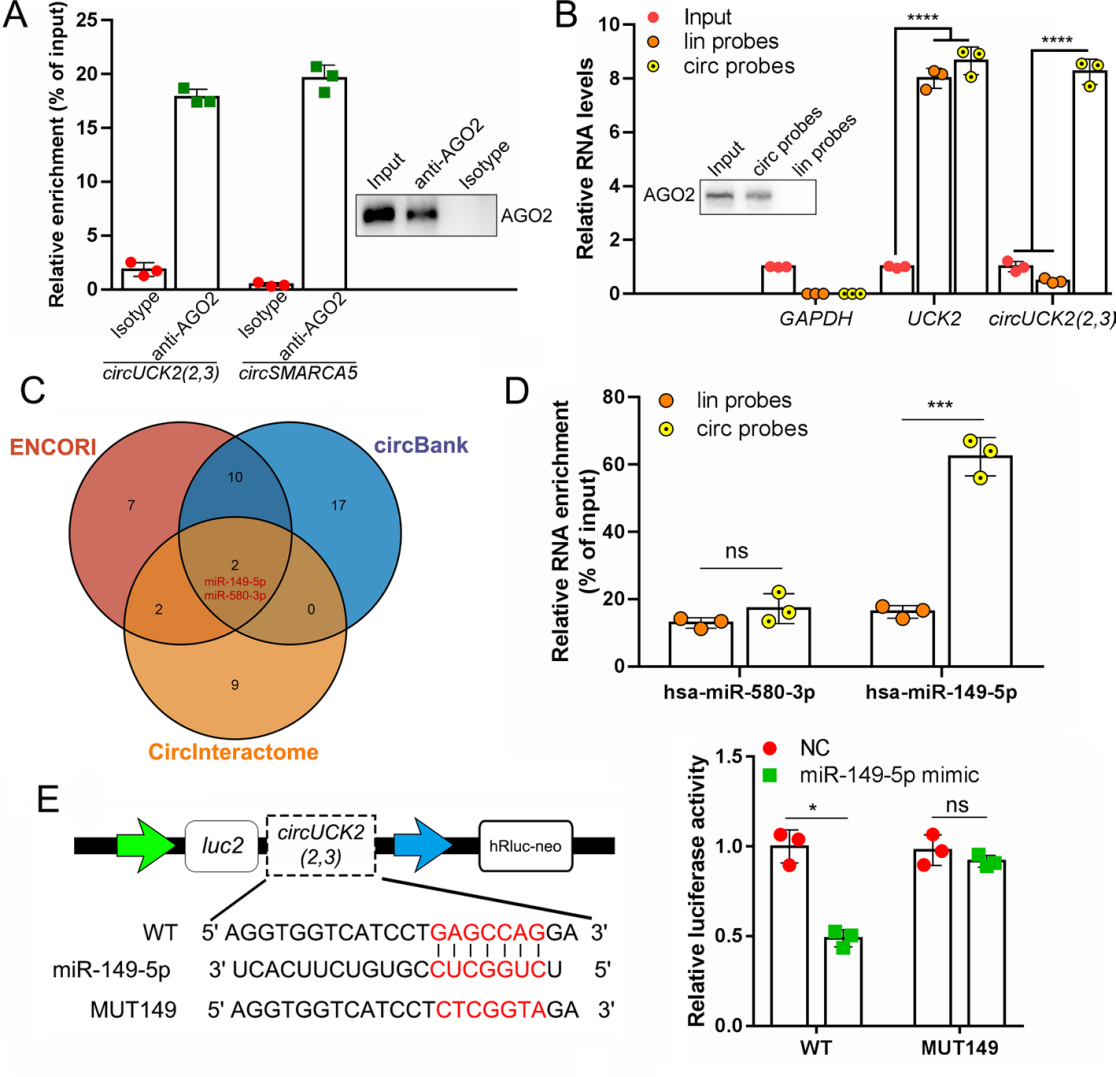
*CircUCK2(2,3)* acts as a sponge of miR-149-5p. **A** AGO2 RIP assay followed by qRT–PCR showed enrichment of *circUCK2(2,3)* and circSMARCA5 in AGO2 precipitates. **B** ChIRP assays demonstrated the enrichment of AGO2 with the *circUCK2(2,3)* precipitates but not with the linear *UCK2*. **C** Combined bioinformatic analysis for putative miRNAs binding with *circUCK2(2,3)*. **D** ChIRP assays followed with Taqman miRNA assays showed the enrichment of miR-149-5p but not miR-580-3p with the *circUCK2(2,3)* precipitates. **E** Dual luciferase assays were performed in 293T cells transfected with miR-149-5p mimics together with reporter vectors carrying WT *circUCK2(2,3)* or *circUCK2(2,3)* mutant with mutated miR-149-5p binding site. Statistical analyses were performed using paired Student’s *t*-tests (NS, no statistical significance; *p < 0.05; ***p < 0.001). *AGO2* argonaute RISC catalytic component 2, *lin probes* linear probes, *circ probes* circRNA probes, *WT* wild type, *MUT149* mutant with mutated miR-149-5p binding site

To validate the interaction between *circUCK2(2,3)* and AGO2, AGO2 RNA immunoprecipitation (RIP) followed by qRT–PCR was performed in PLC/PRF/5 cells. Endogenous *circUCK2(2,3)* and circSMARCA5 (a positive control known to bind AGO2 [36]) were enriched in AGO2 precipitates (Fig. 3A). The interaction between *circUCK2(2,3)* and AGO2 was reciprocally determined by *circUCK2(2,3)* pull down using the ChIRP assay. The *circUCK2(2,3)* pulldown was performed with a pool of circ-probes targeting *circUCK2(2,3)*, and a pool of lin-probes specifically targeting linear *UCK2* as a negative control (Fig. S5E). qRT–PCR showed that lin-probes specifically enriched linear *UCK2* but not *circUCK2(2,3)*, while circ-probes enriched both linear *UCK2* and *circUCK2(2,3)* (Fig. 5B). Western blot assay illustrated that AGO2 co-precipitated with *circUCK2(2,3)* using circ-probes but not lin-probes (Fig. 5B). These findings suggest that *circUCK2(2,3)* physically interacts with AGO2 and may function as a miRNA sponge.

To investigate the downstream target miRNAs of *circUCK2(2,3)*, we conducted bioinformatics analysis using CircInteractome, ENCORI (https://starbase.sysu.edu.cn/) and circBank (http://www.circbank.cn/) databases, which predict interactions between circRNAs and miRNAs. Three databases suggested that *circUCK2(2,3)* might serve as a sponge for miR-149-5p and miR-580-3p (Fig. 3C). The putative binding site of miR-149-5p on *circUCK2(2,3)* is from base 63 to 86, and that of miR-580-3p is from base 166 to 188 (Fig. S5F). A *circUCK2(2,3)* pulldown assay followed by a TaqMan miRNA assay demonstrated miR-149-5p enrichment but not miR-580-3p in *circUCK2(2,3)* precipitates (Fig. 3D), suggesting that *circUCK2(2,3)* serves as a sponge for miR-149-5p. We then constructed luciferase reporters carrying either the wild-type (WT) or the miR-149-5p binding site mutated (MUT149) *circUCK2(2,3)* fragments (Fig. 3E). A luciferase reporter assay showed that miR-149-5p mimics significantly reduced the luciferase activity in the reporter vector carrying WT *circUCK2(2,3)*, not in the one carrying MUT149 *circUCK2(2,3)* (Fig. 3E). We then examined the expressional regulation between *circUCK2(2,3)* and miR-149-5p. Introducing miR-149-5p mimics into PLC/PRF/5 cells or miR-149-5p inhibitors into SK-HEP-1 cells did not affect endogenous *circUCK2(2,3)* level (Fig. S5G). Additionally, overexpression or knockdown of *circUCK2(2,3)* in SK-HEP-1 or PLC/PRF/5 cells, respectively, did not change endogenous miR-149-5p levels (Fig. S5H). This is consistent with previous studies showing no expressional regulatory interaction between circRNAs and their sponging miRNAs [37]. These findings indicate that miR-149-5p is a sponge target of *circUCK2(2,3)*.

### Sponging miR-149-5p is essential for the protumor effects of *circUCK2(2,3)*

By serving as miRNA sponges, circRNAs impede the interactions of miRNAs with their target mRNAs, thereby executing their pathophysiological functions [38–40]. To investigate whether sponging miR-149-5p is essential for the protumor effects of *circUCK2(2,3)*, we introduced miR-149-5p mimics into SK-HEP-1 cells with *circUCK2(2,3)* overexpression, and miR-149-5p inhibitors into PLC/PRF/5 cells with *circUCK2(2,3)* knockdown. MiR-149-5p mimics impaired the protumor effects of *circUCK2(2,3)* on cell proliferation, migration, and invasion in SK-HEP-1 cells (Fig. S6A–C), whereas miR-149-5p inhibitors rescued the tumor repressive effects induced by *circUCK2(2,3)* knockdown in PLC/PRF/5 cells (Fig. S7A–C).

**Fig. 4:**
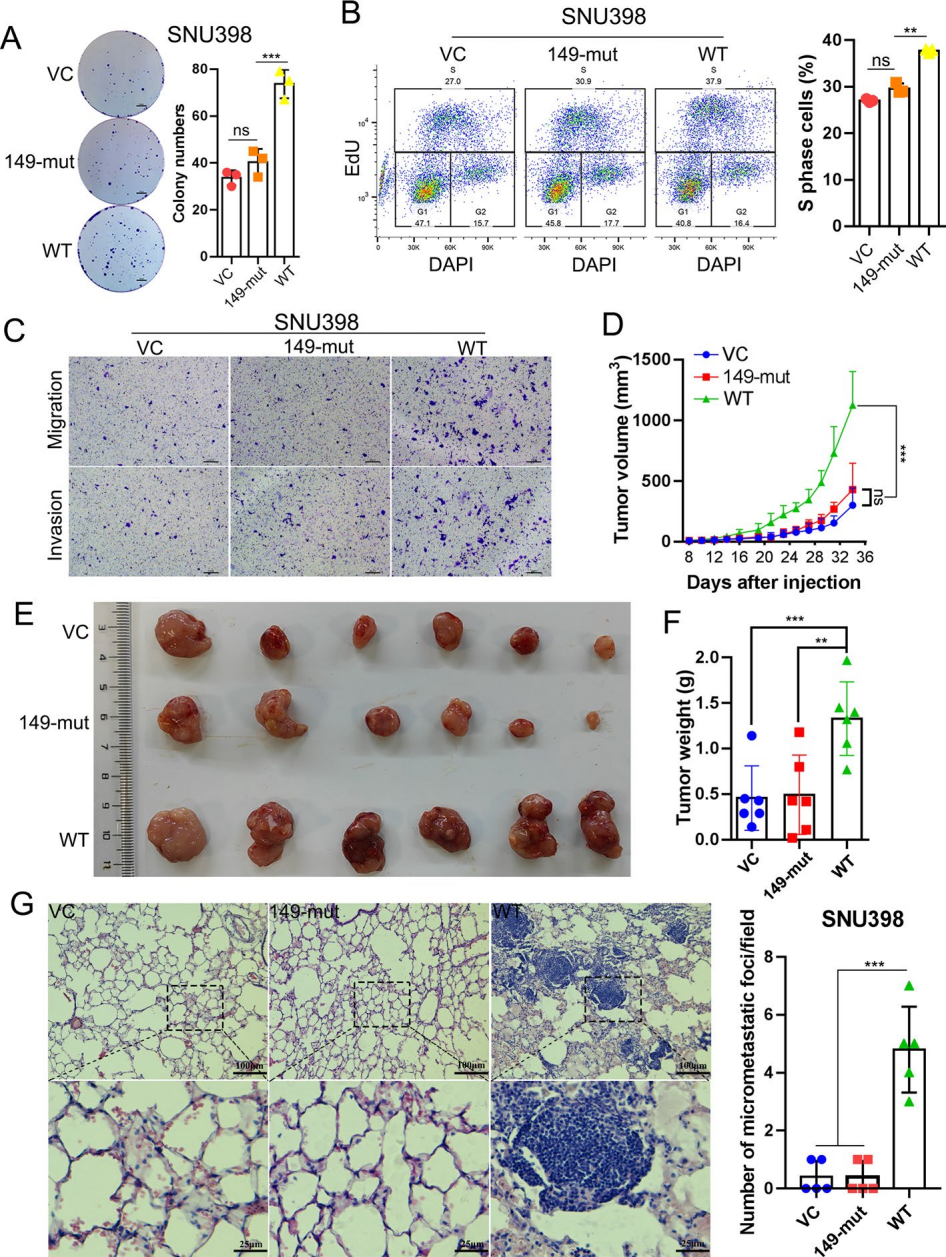
Sponging miR-149-5p is essential for the protumor effects of *circUCK2(2,3)* both in vitro and in vivo. **A** and **B** Colony formation assays (**A**) and EdU flow cytometry assays (**B**) in SNU398 cells with WT *circUCK2(2,3)* or 149-mut *circUCK2(2,3)* overexpression. **C** Transwell migration and invasion assays in SNU398 cells with WT *circUCK2(2,3)* or 149-mut *circUCK2(2,3)* overexpression. **D**–**F** SNU398 cells overexpressing WT *circUCK2(2,3)* or 149-mut were injected into nude mice subcutaneously. Seven days after injection, tumor volumes were measured three times weekly by using a caliper to draw tumor growth curve (**D**) (means ± SD, two-way ANOVA). After 23 days of injection, subcutaneous tumors were dissected and imaged (**E**). Tumor weight comparison among VC, 149-mut, and WT groups (**F**). **G** SNU398 cells with WT *circUCK2(2,3)* or 149-mut overexpression were introduced into nude mice intravenously. Eight weeks after the initial injection, lungs were dissected and lung micrometastases were detected by H&E staining between each group (scale bar, 100 μm and 25 μm). Statistical analyses were performed using paired Student’s *t*-tests (ns, no significance; **p < 0.01; ***p < 0.001). *VC* vector control, *149-mut* miR-149-5p binding site mutant, *WT* wild type, *DAPI* 4′,6-diamidino-2-phenylindole, *EdU* 5-ethynyl-2′-deoxyuridine

To further validate the indispensability of sponging miR-149-5p to the protumor functions of *circUCK2(2,3)*, we constructed an expression vector (named “149-mut”) that expresses *circUCK2(2,3)* with a mutated miR-149-5p binding site. Overexpression of 149-mut was confirmed by qRT–PCR and Sanger sequencing (Fig. S8A and B). Long-term colony formation (Fig. 4A) and CCK8 assays (Fig. S8C) showed that overexpression of 149-mut failed to promote cell proliferation in SNU398 cells. Correspondingly, EdU flow cytometry (Fig. 4B) and EdU staining assays (Fig. S8D) showed an increased S-phase population in cells with WT *circUCK2(2,3)* overexpression but not in cells overexpressing 149-mut. Additionally, transwell migration and invasion assays indicated that, compared to WT *circUCK2(2,3)*, 149-mut failed to promote HCC cell migration and invasion (Fig. 4C). Moreover, xenograft tumor and lung metastasis models in nude mice showed that overexpression of 149-mut had no positive effects on tumor growth and lung metastasis in SNU398 cells in vivo (Fig. 4D–G). These findings strongly indicate that sponging miR-149-5p is essential for the protumor effects of *circUCK2(2,3)*.

### *CNIH4* is a downstream target of the *circUCK2(2,3)*–miR-149-5p axis

**Fig. 5:**
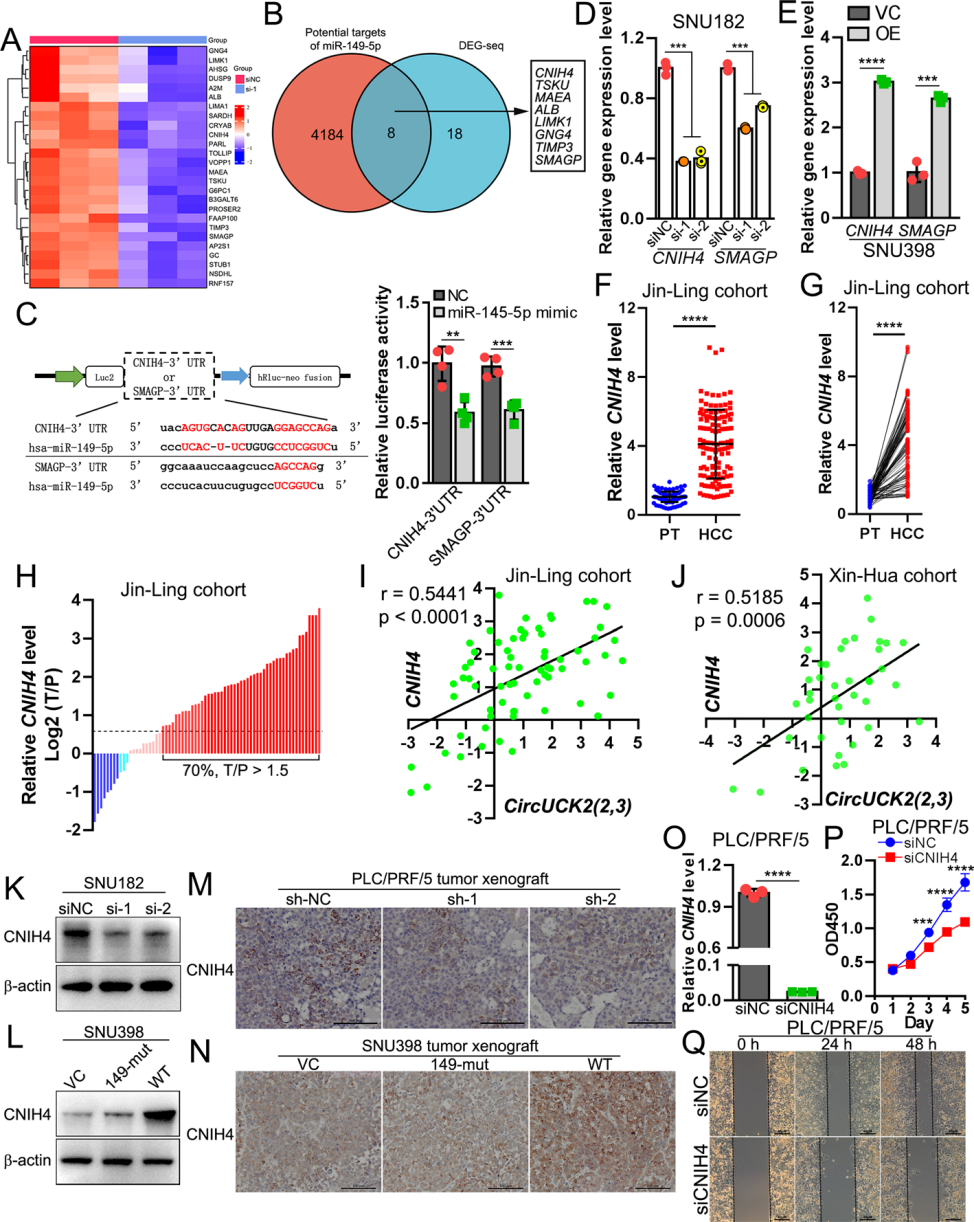
*CNIH4* is a downstream target of the *circUCK2(2,3)*–miR-149-5p axis. **A** Heat map of downregulated genes in PLC/PRF/5 cells with *circUCK2(2,3)* knockdown. **B** Venn plot of downregulated genes and potential miR-149-5p targets. **C** Dual luciferase assays were performed in 293T cells transfected with miR-149-5p mimics together with reporter vectors carrying the 3′UTR of *CNIH4* or *SMAGP*. **D** and **E** qRT–PCR to determine expression changes of *CNIH4* and *SMAGP* in SNU182 cells with *circUCK2(2,3)* downregulation (**D**) and in SNU398 cells with *circUCK2(2,3)* overexpression (**E**). **F** and **G** qRT–PCR analysis of *CNIH4* mRNA levels in 70 paired HCC and peritumor tissues in the Jin-Ling cohort. **H** Tumor versus peritumor expression ratio of *CNIH4* in the Jin-Ling cohort. **I** and **J** Expression correlation between *circUCK2(2,3)* and *CNIH4* in the Jin-Ling cohort (**I**) and in the Xin-Hua cohort (**J**). **K** and **L** Western blotting of CNIH4 protein levels in SNU182 cells with *circUCK2(2,3)* knockdown (**K**) and in SNU398 cells with WT or 149-mut *circUCK2(2,3)* overexpression (**L**). **M** and **N** IHC staining of CNIH4 in PLC/PRF/5 tumor xenografts with *circUCK2(2,3)* knockdown (**M**), and in SNU398 tumor xenografts with WT or 149-mut *circUCK2(2,3)* overexpression (**N**). **O** qRT–PCT to determine knockdown efficiency of *CNIH4*. **P** CCK8 assays in PLC/PRF/5 cells with *CNIH4* knockdown. **Q** Wound healing assays in PLC/PRF/5 cells with *CNIH4* knockdown. Statistical analyses were performed using paired Student’s *t*-tests (**p < 0.01; ***p < 0.001; ****p < 0.0001). *siNC* negative control of siRNA, *si-1* siRNA#1, *si-2* siRNA#2, *siCNIH4* siRNA targeting *CNIH4*, *NC* negative control, *3’UTR* 3' untranslated region, *VC* vector control, *OE* overexpression, *149-mut circUCK2(2,3)* mutant with mutated miR-149-5p binding site, *WT* wild type, *PT* peritumor, *HCC* hepatocellular carcinoma, *luc2* firefly luciferase, *hRluc* renilla luciferase, *neo* neomycin

Previous studies have reported that miR-149-5p functions as a tumor suppressor in HCC by inhibiting the expression of AKT1 [41], MAP2K1 [42], and MTHFR [43, 44]. However, our qRT–PCR results revealed no significant changes in these target genes upon *circUCK2(2,3)* up- or downregulation (Fig. S9A–C). To determine the downstream effectors of the *circUCK2(2,3)*–miR-149-5p axis, we performed RNA-seq in PLC/PRF/5 cells with *circUCK2(2,3)* knockdown. Differential gene expression analysis showed that *circUCK2(2,3)* knockdown resulted in the significant repression of 26 genes (Fig. 5A). After overlapping these 26 differentially expressed genes (DEGs) with 4192 potential targets of miR-149-5p, collected from ENCORI (https://starbase.sysu.edu.cn/) and miRwalk (http://mirwalk.umm.uni-heidelberg.de/), eight common candidates (*CNIH4*, *TSKU*, *MAEA*, *ALB*, *LIMK1*, *GNG4*, *TIMP3*, and *SMAGP*) were selected for further validation (Fig. 5B). Recapitulation of miR-149-5p overexpression by introducing miR-149-5p mimics significantly suppressed the expression *CNIH4* and *SMAGP* (Fig. S9D), whereas suppressing the function of miR-149-5p by introducing miR-149-5p inhibitors significantly induced the expression of *CNIH4* and *SMAGP* (Fig. S9E), suggesting that miR-149-5p may target *CNIH4* and *SMAGP* in HCC cells. Moreover, dual luciferase assays by using reporter vectors carrying the 3′ untranslated region (3′UTR) of either *CNIH4* or *SMAGP* showed that the introduction of miR-149-5p mimics inhibited the luciferase activity (Fig. 5C). Knockdown of *circUCK2(2,3)* resulted in downregulation of *CNIH4* and *SMAGP* in HCC cells (Fig. 5D, and S9F), and overexpression of *circUCK2(2,3)* caused upregulation of *CNIH4* and *SMAGP* in SNU398 (Fig. 5E) and SK-HEP-1 cells (Fig. S9G).

Given the significant upregulation of *circUCK2(2,3)* in HCC tissues (Fig. 1), we assumed that the downstream targets of the *circUCK2(2,3)*–miR-149-5p axis should be similarly induced in HCC. The expression of *CNIH4* in HCC tissues was significantly increased in both cohorts (Fig. 5F–H, the Jin-Ling cohort; S10A to C, the Xin-Hua cohort). Marked upregulation of *CNIH4* (fold change, T/P > 1.5) was detected in 70% and 60% of HCC samples in the Jin-Ling (Fig. 5H) and the Xin-Hua cohorts (Fig. S10C), respectively. Additionally, *CNIH4* upregulation in HCC tissues was confirmed in the TCGA–LIHC dataset (Fig. S10D). However, no significant expression difference of *SMAGP* was detected between HCC and peritumor tissues (Fig. S10E and F). Expression correlation analysis showed significantly positive correlation between *circUCK2(2,3)* and *CNIH4* in both HCC cohorts (Fig. 5I, J). Furthermore, Kaplan–Meier analysis in the TCGA–LIHC dataset showed that the expression of *CNIH4* is negatively associated with overall survival (Fig. S10G) and recurrence-free survival (Fig. S10H) in HCC patients.

Additionally, western blotting assays showed a decrease in CNIH4 protein level in SNU182 cells with *circUCK2(2,3)* knockdown (Fig. 5K), and an increase in CNIH4 expression in SNU398 cells with overexpression of WT *circUCK2(2,3)*, but not 149-mut (Fig. 5L). Moreover, IHC staining of CNIH4 in tumor xenografts showed reduced CNIH4 protein expression in PLC/PRF/5 tumor xenografts with *circUCK2(2,3)* knockdown (Fig. 5M), and increased CNIH4 staining in SNU398 tumor xenografts with WT but not 149-mut *circUCK2(2,3)* overexpression (Fig. 5N). Functionally, *CNIH4* knockdown (Fig. 5O) has been experimentally demonstrated to impair proliferation and migration in PLC/PRF/5 cells (Fig. 5P, Q). These findings suggest that *CNIH4* is a downstream target of the *circUCK2(2,3)*–miR-149-5p axis.

### *CircUCK2(2,3)* activates TGFα–EGFR signaling and downstream AKT and ERK

**Fig. 6:**
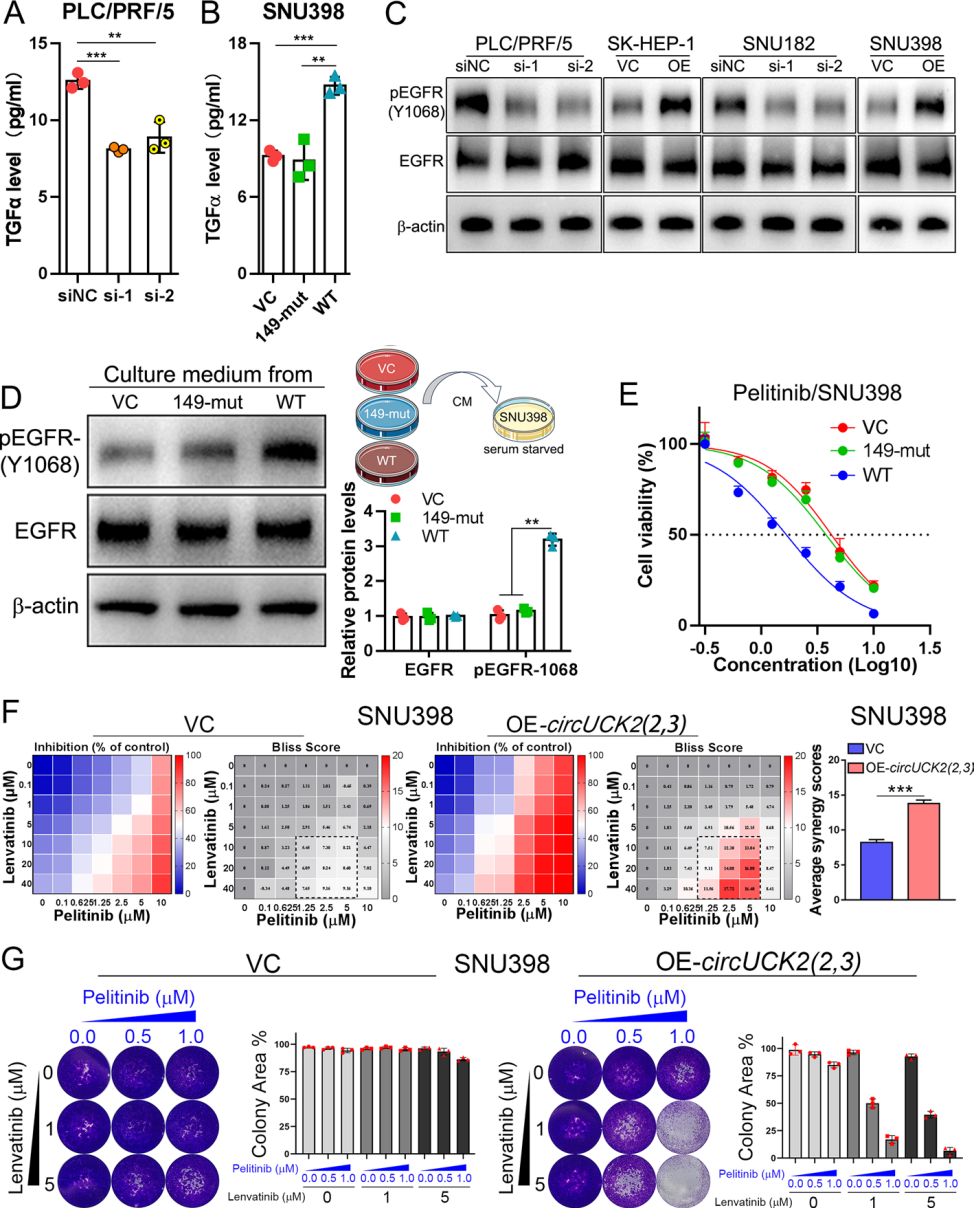
*CircUCK2(2,3)* increases secreted TGFα and subsequent EGFR activation, and sensitizes HCC cells to EGFR inhibitors and the synergistic cytotoxic effect of lenvatinib combined with EGFR inhibitors. **A** and **B** ELISA assays of TGFα in PLC/PRF/5 with *circUCK2(2,3)* knockdown (**A**) or in SNU398 with WT or 149-mut *circUCK2(2,3)* overexpression (**B**). **C** Protein levels of pEGFR-(Y1068) and EGFR in HCC cells with *circUCK2(2,3)* knockdown or overexpression. **D** Schematic showing the procedure of medium transfer assay. Protein levels of pEGFR-(Y1068) and EGFR in SNNU398 cells treated with culture medium from SNU398 cells with WT or 149-mut *circUCK2(2,3)* overexpression. **E** Cytotoxic effects of pelitinib in SNU398 cells overexpressing WT or 149-mut *circUCK2(2,3*). **F** and **G** A bliss independent model to evaluate the synergistic killing of lenvatinib with pelitinib in SNU398 cells with or without *circUCK2(2,3)* overexpression in short-term viability assays and long-term clonogenic assays. Synergyfinder.org web tool was used to calculate the Bliss synergy scores. Average synergy scores for the indicated drug combinations were calculated. Statistical analyses were performed using paired Student’s *t*-tests (**p < 0.01; ***p < 0.001). *siNC* siRNA negative control, *si-1* siRNA#1, *si-2* siRNA#2, *VC* vector control, *OE* overexpression, *149-mut circUCK2(2,3)* mutant with mutated miR-149-5p binding site, *WT* wild type, *CM* culture medium, *IC*_*50*_ half-maximal inhibitory concentration

Given that CNIH4 functions as a cargo receptor for TGFα transport and secretion, we then examined whether *circUCK2(2,3)* affects TGFα secretion. ELISA assays showed that *circUCK2(2,3)* knockdown reduced TGFα secretion in PLC/PRF/5 cells (Fig. 6A), and overexpression of WT *circUCK2(2,3)* but not 149-mut enhanced TGFα secretion in SNU398 (Fig. 6B). Since TGFα is a high-affinity ligand of EGFR, we then examined the EGFR activation under conditions of *circUCK2(2,3)* knockdown or overexpression. HCC cells with *circUCK2(2,3)* knockdown showed decreased EGFR activation, whereas HCC cells with *circUCK2(2,3)* overexpression displayed increased EGFR activation (Fig. 6C). Moreover, culture medium of SNU398 stable transfectants overexpressing WT *circUCK2(2,3)* but not 149-mut markedly induced EGFR activation (Fig. 6D).

Given that enhanced EGFR phosphorylation activates multiple downstream signaling pathways, including the MAPK-ERK, the PI3K-AKT, the JAK-STAT3, and the NF-κB [45–48], all of which play critical roles in cancer cell proliferation and migration, we investigated the activation of these pathways in response to *circUCK2(2,3)* knockdown or overexpression. Western blot analysis revealed that downregulation or overexpression of *circUCK2(2,3)* respectively decreased or increased pERK and pAKT levels in HCC cells, without affecting p-p65 (NF-κB) and pSTAT3 (Fig. S11A), These results suggest that *circUCK2(2,3)*-induced EGFR phosphorylation activates the downstream MAPK-ERK and PI3K-AKT signaling pathways, but does not affect JAK-STAT3 or NF-κB pathways.

In line with these findings, CCK-8 assays showed that treatment with selumetinib (a MEK inhibitor), ASN007 (an ERK inhibitor), and perifosine (an AKT inhibitor), but not stattic (a STAT3 inhbitor), significantly impaired *circUCK2(2,3)*-induced proliferation in SNU398 cells (Fig. S11B). Furthermore, western blot analysis demonstrated that treatment with selumetinib, ASN007, and perifosine, but not stattic, reversed the changes in EMT markers induced by *circUCK2(2,3)* (Fig. S11C).

Transwell migration and invasion assays revealed that treatment with selumetinib and ASN007 significantly inhibited *circUCK2(2,3)*-induced cell migration and invasion in SNU398 cells (Fig. S11D). Intriguingly, although perifosine reversed *circUCK2(2,3)*-induced EMT marker changes, it failed to inhibit the enhanced migration and invasion induced by *circUCK2(2,3)* (Fig. S11D). This suggests that changes in EMT marker expression do not fully reflect the migration and invasion capabilities of cancer cells.

Together, these findings indicate that *circUCK2(2,3)*-induced EGFR phosphorylation could activate downstream MAPK-ERK and PI3K-AKT signaling, which contribute to enhanced cell proliferation, migration and invasion.

### *CircUCK2(2,3)* sensitizes HCC cells to the synergistic cytotoxicity of lenvatinib and EGFR inhibitors

Given that *circUCK2(2,3)* can induce TGFα-mediated EGFR activation, we then speculated that *circUCK2(2,3)* may, in turn, affect the response of HCC cells to EGFR inhibitors. CCK-8 assays showed that *circUCK2(2,3)* overexpression sensitized HCC cells to multiple EGFR inhibitors, including gefitinib, vandetanib and pelitinib (Fig. 6E, Fig. S12G to I), whereas *circUCK2(2,3)* knockdown showed no or marginal effects (Fig. S12A–D). Previous studies have demonstrated that EGFR activation confers HCC lenvatinib resistance [49–51]. To examine whether *circUCK2(2,3)* affects the cytotoxicity of lenvatinib in HCCs, we conducted lenvatinib cytotoxicity assays in HCC cells with *circUCK2(2,3)* overexpression or knockdown. Although *circUCK2(2,3)* can activate EGFR, neither overexpression nor knockdown of *circUCK2(2,3)* affected the response to lenvatinib in HCC cells (Fig. S12E and F), suggesting that the underlying mechanism of EGFR-induced lenvatinib resistance is complicated.

Since the combination of EGFR inhibitors and lenvatinib is synergistically lethal to HCC cells, we then tested whether *circUCK2(2,3)* can affect this synergistic killing effect by treating SNU398 cells with or without *circUCK2(2,3)* overexpression. Synergy matrices combining lenvatinib with gefitinib, vandetanib, or pelitinib showed higher cytotoxicities in SNU398 cells with *circUCK2(2,3)* overexpression compared to cells without *circUCK2(2,3)* overexpression, both in short-term viability assays and long-term clonogenic assays (Fig. 6F, G, and Fig. S13), suggesting that *circUCK2(2,3)* may enhance the synergistic lethal effects of lenvatinib and EGFR inhibitors in HCC cells.

**Fig. 7:**
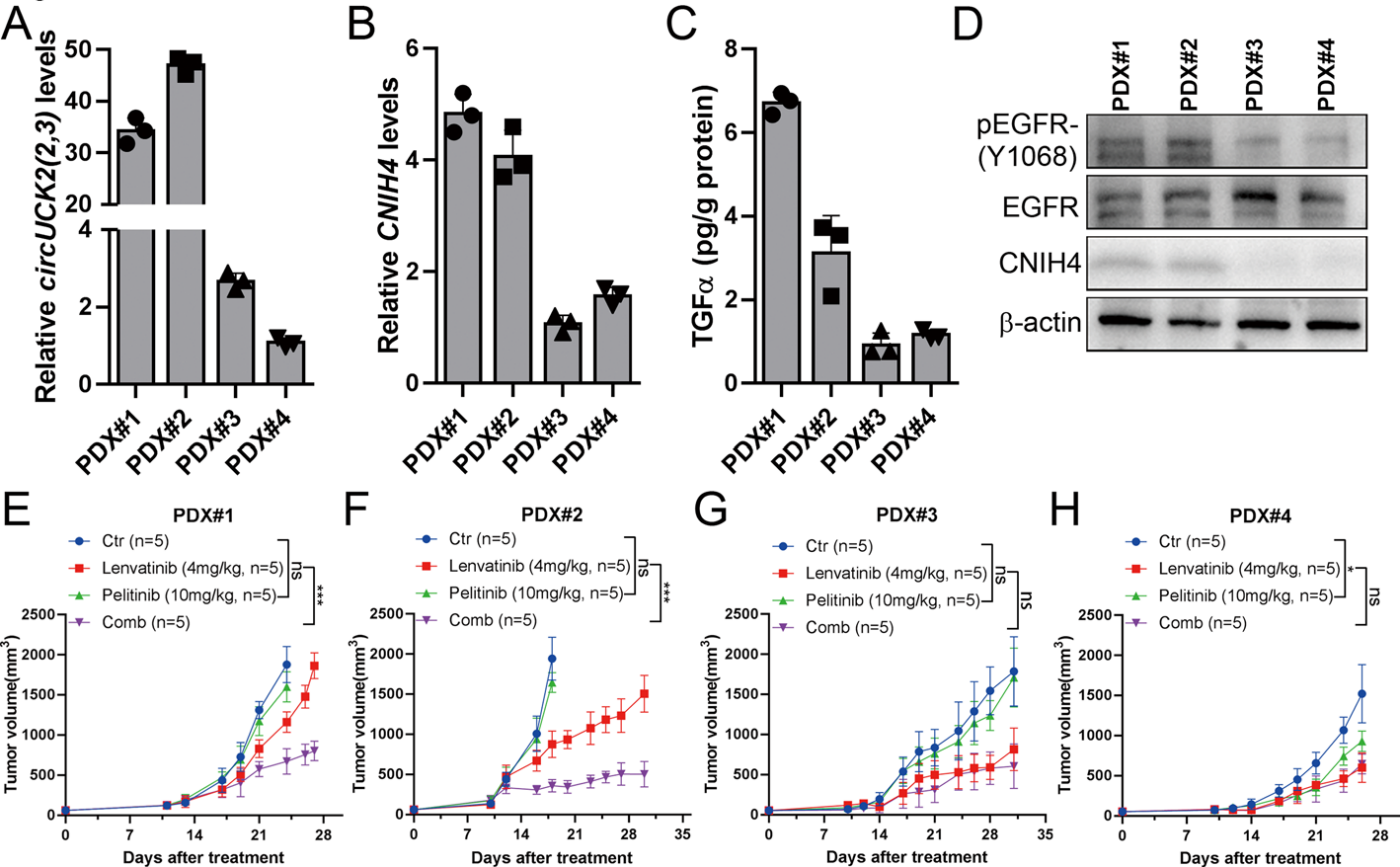
*CircUCK2(2,3)* sensitizes patient-derived HCC xenografts to the synergistic cytotoxic effect of lenvatinib with pelitinib. **A** and **B** qRT–PCR to determine the endogenous *circUCK2(2,3)* (**A**) and *CNIH4* (**B**) levels in four HCC PDXs. **C** ELISA assays of TGFα in lysates of four HCC PDXs. **D** Protein levels of pEGFR-(Y1068), EGFR, and CNIH4 in four HCC PDXs. **E**–**H** Growth curves of four HCC PDXs in mice treated with vehicle control, lenvatinib (4 mg/kg), pelitinib (10 mg/kg), or their combination (lenvatinib, 4 mg/kg plus pelitinib, 10 mg/kg). *n* = 5 mice per group. Data are mean ± SD. Statistical analyses were performed using by two-way ANOVA. *P*-values were determined by two-way ANOVA with Sidak’s multiple comparisons (ns, *p* > 0.05; *p < 0.05; ***p < 0.001)

We next investigated this effect in four patient-derived xenograft (PDX) mouse models. qRT–PCR showed higher *circUCK2(2,3)* and *CNIH4* expression in PDX#1 and PDX#2 compared to PDX#3 and PDX#4 (Fig. 7A, B). In line with our findings in HCC cell lines, PDXs with higher *circUCK2(2,3)* expression showed greater CNIH4, phosphorylated EGFR, and secreted TGFα levels compared to PDXs with low *circUCK2(2,3)* expression (Fig. 7C, D). Given that the combination of lenvatinib with pelitinib displayed the most significant synergistic killing effect in the long-term clonogenic assays (Fig. 6G), we then chose combining lenvatinib with pelitinib to treat these PDXs. Consistent with our in vitro findings, compared to lenvatinib treatment alone, the drug combination elicited greater tumor regression in two *circUCK2(2,3)*^high^ PDXs (PDX#1 and #2) (Fig. 7E, F), but showed less synergistic killing effect in two *circUCK2(2,3)*^low^ PDXs (PDX#3 and #4) (Fig. 7G, H).

Given that *circUCK2(2,3)*-mediated EGFR activation leads to downstream activation of AKT and ERK signaling, which in turn enhances HCC cell proliferation, migration, and invasion, we next examined whether combined treatment with lenvatinib and pelitinib could impact these pathways. Overexpression of *circUCK2(2,3)* significantly induced the activation of EGFR, AKT and ERK (Fig. S14A). Combined treatment with lenvatinib with pelitinib resulted in a more substantial inhibition on pAKT and pERK compared to either drug alone (Fig. S14B and C). However, this combination had no effect on p-p65 and pSTAT3 (Fig. S14D and E), suggesting that the synergistic effects of lenvatinib and pelitinib are primarily due to their enhanced inhibition on AKT and ERK signaling.

Collectively, these findings suggest that *circUCK2(2,3)* can activate TGFα–EGFR signaling, and sensitize HCC to the synergistic cytotoxic effect of lenvatinib and EGFR inhibitors.

### Characteristics of the biosynthesis regulators of *circUCK2(2,3)*

Given a weak expression correlation between *circUCK2(2,3)* and linear *UCK2* (Fig. S1L and M), we speculated the existence of independent elements specifically for *circUCK2(2,3)* transcription. We found a chromatin region upstream of *circUCK2(2,3)* containing a cluster of DNase I hypersensitivity sites (Fig. S15A, marked by a red dashed rectangle), suggesting that this chromatin region may be accessible and serve as a putative promoter for *circUCK2(2,3)*. Consistent with this, we observed enrichment of both H3K4me3 and H3K27Ac, chromatin markers of active promoter [52, 53], in this region (Fig. S15A, marked by a red dashed rectangle). A series of fragments located at or flanking this chromatin region were then cloned into a pGL3-basic plasmid for promoter activity analysis (Fig. S15B). Plasmids carrying fragments from −2300 to −1 or from −1300 to −1 showed no luciferase activity, whereas deletion of nucleotides from −1300 to −1000 resulted in an over tenfold increase in luciferase activity (Fig. S15B). Sequence deletion from −90 to −1 eliminated luciferase activity (Fig. S15B). These findings suggest that a core promoter between −90 and −1, and a repressive element between −1300 and −1000 are located upstream of *circUCK2(2,3)*.

To further explore the transcriptional regulation of *circUCK2(2,3)*, we employed PROMO software and identified six putative transcriptional factors (TFs), including C/EBPbeta, TFII-I, YY1, GR-alpha, AP-2alphaA, and TFIID, at the core promoter region (−90 to −1) of *circUCK2(2,3)* with the highest template dissimilarity (0%) [54] (Fig. S15C). Deletion of the TFII-I binding element greatly impaired the luciferase activity, whereas deletion of the YY1 binding element significantly enhanced the luciferase activity (Fig. S15C), suggesting that binding of TFII-I or YY1 to the core promoter may stimulate or suppress *circUCK2(2,3)* transcription, respectively. Furthermore, knockdown of TFII-I significantly repressed *circUCK2(2,3)* expression (Fig. S15D and E), whereas knockdown of *YY1* had no significant effect on *circUCK2(2,3)* expression in PLC/PRF/5 cells (Fig. S15F and G). Moreover, overexpression of TFII-I enhanced the expression of endogenous *circUCK2(2,3)*, but not the host *UCK2* gene (Fig. S15H and I).

Previous studies have demonstrated that circRNA back-splicing is commonly regulated by flanking intronic complementary sequences (ICSs) [55, 56], with *Alu* element the most prevalent ICSs promoting circRNA back-splicing in primates [56–58]. Two *Alu* elements (*AluJb* and *AluSx*) are located in intron 1, and one *Alu* element (*AluJb*) is located in intron 3 flanking *circUCK2(2,3)* (Fig. S15A, and S16A). Interestingly, the intron 1 *AluJb* and *AluSx* are in antisense orientation, whereas the intron 3 *AluJb* is in sense orientation (Fig. S15A, and S16A), suggesting that these inverted *Alu* elements may form complementary base pairing to regulate the generation of *circUCK2(2,3)*. To investigate this, we designed sgRNAs aim to specifically remove the intron 1 *AluJb* and *AluSx,* or the intron 3 *AluJb* (Fig. S16A). PLC/PRF/5 clones with heterozygous or homozygous deletion of the intron 1 *AluJb* and *AluSx,* or the intron 3 *AluJb* were established, as evidenced by genotyping and Sanger sequencing (Fig. S16B and D). The removal of these ICIs resulted in a marked decrease in *circUCK2(2,3)* expression, but did not affect the expression of the host *UCK2* in PLC/PRF/5 cells (Fig. S16C and E), suggesting that these flanking *Alu* elements are specific for *circUCK2(2,3)* production.

Collectively, these findings suggest that the expression of *circUCK2(2,3)* is regulated by an upstream 90 nt core promoter, a transcriptional factor TFII-I, and the ICSs flanking *circUCK2(2,3)*.

## Discussion

CircRNAs and their linear host mRNAs are respectively back-spliced and canonically spliced from the same pre-mRNAs, suggesting that they may be expressed in a competitive splicing mode, and their expression should theoretically be negatively correlated. This would be true if the production of circRNAs is solely regulated by back-splicing efficiency. In reality, various factors can affect circRNA expression, including back-splicing efficiency, Pol II transcription elongation rate (TER), the existence of ICSs, spliceosomes, RNA-binding proteins, and epigenetic modifications [59, 60]. Under the influence of all these circRNA-specific regulatory factors, the expression correlation between circRNAs and their host genes is typically weak [61]. Consistent with this, we also found weak correlation between *circUCK2(2,3)* and *UCK2*. This is at least partly due to the regulatory effects of flanking ICSs and the promoter of *circUCK2(2,3)*.

The function of miR-149-5p has been evaluated in various diseases, including cancer. Although both oncogenic and tumor-suppressive roles of miR-149-5p have been reported [62], miR-149-5p functions as a tumor suppressor in most common cancers, including breast cancer [63], lung cancer [64], gastric cancer [65], colorectal cancer [66, 67], and thyroid carcinoma [68]. In HCC, miR-149-5p has been reported to inhibit tumor progression. Through bioinformatic analysis, ChIRP assay, and luciferase assay, we validated the direct binding between *circUCK2(2,3)* and miR-149-5p. Rescue assays illustrated that miR-149-5p mimics or inhibitors respectively reversed the stimulatory or inhibitory effects of *circUCK2(2,3)* overexpression or knockdown in HCC cells. More importantly, we further demonstrated that the *circUCK2(2,3)* mutant with mutated miR-149-5p biding site failed to promote in vitro cell proliferation, migration, and invasion, as well as in vivo tumor growth and metastasis. Therefore, these findings strongly suggest that *circUCK2(2,3)* sponges miR-149-5p to play a tumor-promoting role in HCC.

CNIH4, a member of the CORNICHON family, plays an evolutionarily conserved role in TGFα transport and secretion [20–22], as well as in intracellular trafficking of G protein-coupled receptors (GPCRs) from endoplasmic reticulum (ER) to plasma membrane [69]. Although enriched CNIH4 expression is observed in mouse germ cells, genetic deletion of *Cnih4* does not affect mouse gametogenesis and fertility [70]. The role of CNIH4 in HCC and its underlying mechanism have not been explored. We demonstrated that the expression of *CNIH4* in HCC tissues is upregulated and positively correlated with that of *circUCK2(2,3)*. High *CNIH4* expression is associated with lower overall survival and disease-free survival in HCC patients, Furthermore, downregulation of *CNIH4* inhibited cell proliferation and migration in HCC cells, suggest that *CNIH4* may play a significant role in HCC progression and could be a potential therapeutic target.

TGFα is a membrane-bound or soluble ligand of EGFR. A previous study has reported that hepatitis B virus (HBV) DNA can induce TGFα expression [71], which, at least in part, explains the elevated TGFα levels in HCC patients [72]. Overexpression of TGFα or activation of TGFα signaling has been closely associated with HCC initiation and progression. For instance, spontaneous HCC development has been observed in transgenic mice with human TGFα overexpression [73]; co-expression of TGFα greatly accelerates HCC formation in c-Myc transgenic mice [74]. Although both TGFα and EGF are high-affinity ligands of EGFR [75], elevated secretion of TGFα was observed in 65% of HCC patients, whereas only 16% of HCC patients showed increased EGF secretion [76], suggesting that TGFα may play a major role in EGFR signaling activation in HCC. In this study, we demonstrated that, by sponging miR-149-5p, *circUCK2(2,3)* releases *CNIH4* from miR-149-5p-mediated inhibition, subsequently increasing TGFα secretion and EGFR activation. Meanwhile, TGFα-induced EGFR activation has been reported to promote tumor progression in cervical cancer [77], ovarian cancer [78], and breast cancer [79]. Therefore, it is worth investigating the presence of this regulatory interaction linking *circUCK2(2,3)* and TGFα in these cancer types.

The activation of EGFR can trigger the downstream activation of multiple signaling pathways, including MAPK-ERK, PI3K-AKT, JAK-STAT3, and NF-κB [45–48]. In this study, we observed that *circUCK2(2,3)-*mediated EGFR activation led to the activation of ERK and AKT signaling, but not STAT3 and NF-κB signaling. This is consistent with numerous previous studies showing that EGFR activation typically leads to the activation of ERK and AKT. The differential activation of downstream EGFR signaling pathways is not unusual and can be influenced by several factors, including the specific ligand binding to EGFR, the cellular context, and the presence of coreceptors or scaffolding proteins. For instance, EGFR-dependent STAT3 activation requires the association of the IL-6 receptor and gp130 with EGFR [46]. EGFR induced activation of NF-κB depends on the presence of the IKK complex, which includes both catalytic subunits IKKα and IKKβ, as well as the scaffold protein NEMO/IKKγ [45]. The inability of *circUCK2(2,3)* to induce STAT3 and NF-κB activation may suggest that these essential EGFR cofactors are absent or not functional in HCC cells.

Lenvatinib, while effective as a first-line treatment for unresectable hepatocellular carcinoma (HCC), has limitations such as potential resistance, significant side effects, and limited efficacy in certain patient subpopulations [80]. Recent studies suggest that combining lenvatinib with EGFR inhibitors could enhance therapeutic efficacy for HCC treatment [49]. A clinical trial by Jin et al. showed improved antitumor activity and manageable safety profiles with lenvatinib–gefitinib in lenvatinib-resistant HCCs [49]. In this study, we demonstrated that *circUCK2(2,3)* overexpression enhances the synergistic killing effects of lenvatinib when combined with multiple EGFR inhibitors in HCC cells and in PDX clinical models, suggesting that tumor *circUCK2(2,3)* levels may present a promising biomarker predicting the response to combined treatment of lenvatinib and EGFR inhibitors.

Our results differ from those of a recent study showing a direct regulatory interaction between the expression of *circUCK2(2,3)* and *UCK2* [81]. While their findings are interesting and somewhat counterintuitive, given that previous studies demonstrating that the increased *UCK2* expression in HCC is associated with *UCK2* gene hypomethylation and copy number amplification [82, 83]. Additionally, we demonstrated a marginal expression correlation between *circUCK2(2,3)* and *UCK2* in our two independent HCC cohorts (Fig. S1L and M), and observed unchanged endogenous *UCK2* expression levels upon knockdown, knockout, or overexpression of *circUCK2(2,3)* (Fig. S2D and E).

## Conclusions

Collectively, our study primarily demonstrates that *circUCK2(2,3)* is upregulated in HCC tissues and serves as an independent risk factor for RFS in HCC patients. By acting as a sponge for miR-149-5p, *circUCK2(2,3)* alleviates the inhibitory effect of miR-149-5p on *CNIH4*, leading to increased TGFα secretion and subsequent EGFR activation. This action promotes HCC progression on the one hand, but on the other hand, it sensitizes HCC cells to EGFR inhibitors and enhances the synergistic killing effects of lenvatinib combined with EGFR inhibitors. Therefore, our findings uncover a new molecular mechanism of HCC progression and suggest that *circUCK2(2,3)* serves not only as a promising therapeutic target, but also as a biomarker for precision medicine in HCC treatment.

## Supplementary Information

Supplementary material 1.

Supplementary material 2.

## Data Availability

The circRNA-seq and RNA-seq data have been deposited into the GEO repository with accession number GSE263134 and GSE263915, respectively. Additional data that support the findings of this study are available from the corresponding author upon reasonable request.

## Abbreviations

149-mut: *CircUCK2(2,3)* mutant with a mutated miR-149-5p binding site
3′UTR: 3′ Untranslated region
AGO2: Argonaute RISC catalytic component 2
AKT1: AKT serine/threonine kinase 1
ALB: Albumin
BIRC5: Baculoviral IAP repeat-containing 5
CCK8: Counting kit-8
CCR5: C–C motif chemokine receptor 5
C/EBPbeta: CCAAT/enhancer-binding protein beta
ChIRP: Chromatin isolation by RNA purification
CircInteractome: CircRNA interactome
CircRNA: Circular RNA
CIRI: CircRNA identifier
CM: Culture medium
CNIH4: Cornichon family AMPA receptor auxiliary protein 4
CNV: Copy number variation
COSMIC: Catalogue of somatic mutations in cancer
CPAT: Candidate physical ability test
CPC2: Coding potential calculator v2.0
DAPI: 4′,6-Diamidino-2-phenylindole
DEGs: Differentially expressed genes
DMEM: Dulbecco’s modified Eagle medium
Edu: 5-Ethynyl-2′-deoxyuridine
EGFR: Epidermal growth factor receptor
ENCORI: Encyclopedia of RNA Interactomes
ER: Endoplasmic reticulum
FBS: Fetal bovine serum
GAPDH: Glyceraldehyde-3-phosphate dehydrogenase
GNG4: G protein subunit gamma 4
GPCRs: G protein-coupled receptors
GR-alpha: Glucocorticoid receptor alpha
H3K4me3: Tri-methylation of lysine 4 on histone H3
H3K27Ac: Acetylated histone H3 lysine 27
HCC: Hepatocellular carcinoma
ICIs: Immune checkpoint inhibitors
ICSs: Intronic complementary sequences
LIMK1: LIM domain kinase 1
MAEA: Macrophage erythroblast attacher, E3 ubiquitin ligase
MALAT1: Metastasis associated lung adenocarcinoma transcript 1
MAP2K1: Mitogen-activated protein kinase kinase 1
MTHFR: Methylenetetrahydrofolate reductase
PDX: Patient-derived xenograft
qRT–PCR: Quantitative real-time PCR
RIP: RNA immunoprecipitation
RPMI: Roswell Park Memorial Institute
SMAGP: Small cell adhesion glycoprotein
TCGA–LIHC: The Cancer Genome Atlas Liver Hepatocellular Carcinoma
TFIID: Transcription factor IID
TGFα: Transforming growth factor alpha
TIMP3: TIMP metallopeptidase inhibitor 3
TKIs: Tyrosine kinase inhibitor
TSKU: Tsukushi, small leucine-rich proteoglycan
UCK2: Uridine-cytidine kinase 2
WT: Wild-type
YY1: YIN-YANG-1
