# Supplementary material for: CircUCK2(2,3) promotes cancer progression and enhances synergistic cytotoxicity of lenvatinib with EGFR inhibitors via activating CNIH4–TGFα–EGFR signaling
Source: Cell Mol Biol Lett. 2025 Jan 30;30:15. doi: 10.1186/s11658-025-00690-1 (PMC11781035; doi:10.1186/s11658-025-00690-1)
Supplement: Supplementary file 1 — Supplementary material 1. [file 11658_2025_690_MOESM1_ESM.docx]

***CircUCK2(2,3)* Promotes Cancer Progression and Enhances Synergistic Cytotoxicity of Lenvatinib with EGFR Inhibitors via Activating CNIH4-TGFα-EGFR Signaling**

**Supplementary file**

**Supplementary Materials and Methods**

**Plasmid construction and RNA interference (RNAi)**

For the construction of WT or 149-mut *circUCK2(2,3)* overexpression vectors, the DNA fragments of WT or 149-mut *circUCK2(2,3)* were cloned into the pLC5-ciR vector (Geneseed Biotech, Guangzhou, China) between EcoR1 and BamH1 sites. For the construction of dual luciferase reporter vectors, the DNA fragments of WT or 149-mut *circUCK2(2,3)*, 3’UTRs of *CNIH4* or *SMAGP* were cloned into the pmirGLO Dual-Luciferase miRNA Target Expression Vector (Promega, Beijing, China) between Xho1 and Xba1 sites. For the construction of shRNA expression vectors, shRNAs specifically knocking down *circUCK2(2,3)* were cloned into the pLV3(H1/GFP&Puro) vector (GenePharma, Shanghai, China). For the construction of luciferase reporter vectors detecting the transcriptionally regulatory elements of *circUCK2(2,3)*, the DNA fragments upstream of *circUCK2(2,3)* at indicated lengths were cloned into pGL3-Basic vector between Kpn1 and Xho1 sites. For the construction of TFII-I expression vector, the Flag-tagged CDS region of TFII-I was cloned into pcDNA3.1(+) vector between the Nhe1 and Not1 sites. For the construction of sgRNA expression vector, annealed sgRNA oligos were cloned into pL-CRISPR.EFS.tRFP (addgene: #57819) between Esp3I sites. siRNAs targeting *circUCK2(2,3), CNIH4,* TFII-I and *YY1* (GenePharma, Shanghai, China) were used for transient knockdown of indicated genes. The sequence information of primers, siRNAs, shRNAs, and sgRNA oligos was provided in Supplementary table 3 and 4.

**qRT-PCR**

Total RNA was extracted using TRIzol (Life Technologies, USA) in accordance with the manufacturer's instructions. Reverse transcription was carried out according to the manufacturer's instructions (Toyobo, Japan). cDNA for circular RNA was reverse transcribed with random primer (Takara bio, cat#3802), dNTP (Thermo Scientific, R0192), Maxima Reverse Transcriptase (Thermo Scientific, EP0742) and Ribolock RNase Inhibitor (Thermo Scientific, EO0381). The primers used in this study were designed using the Primer-BLAST tool provided by [www.ncbi.nlm.nih.gov](http://www.ncbi.nlm.nih.gov). The sequence information of primers was provided in Supplementary Table 3. qRT-PCR was conducted on a (QuantStudio™ 1 Plus Real-Time PCR System) with TB Green Premix Ex Taq (Takara, R420A). 18S RNA or *GAPDH* was used as internal control for quantification of circRNAs or mRNA, respectively. miRNA levels were measured by TaqMan miRNA assays (Thermo Scientific, USA). The relative expression of genes was calculated by 2^–ΔΔCT^.

**RNase R assay**

Total RNAs (2 μg) were first subjected to RNase R (4U/μg) (Biosearch Technologies, E0111-20D) or mock treatment at 37°C for 30 min, and subsequently purified by miRNeasy Mini Kit (QIAGEN, USA). 500 ng purified RNA was used for reverse transcription. RT-PCR was performed to determine the levels of *circUCK2(2,3)*, linear *UCK2*, and *GAPDH.*

**Actinomycin D assay**

Cells (4x10^5^/well) were seeded at a 6-well plate and treated with Actinomycin D (2μg/ml) (Selleckchem, China). Total RNAs of treated cells were isolated at indicated time points. Expression level changes of endogenous *circUCK2(2,3)* and linear *UCK2* before or after Actinomycin D treatment were determined by qRT-PCR.

**RNAscope in situ hybridization (ISH)**

RNAscope assay was used to determine the intracellular distribution of *circUCK2(2,3)*. Customized double Z oligonucleotide probes target *circUCK2(2,3)* (Cat#1101651-1, ACD), BaseScope positive control probe-Hs-PPIB-1ZZ (Cat#710171, ACD), BaseScope negative control probe-DapB-1ZZ (Cat#701021, ACD) and RNA Scope Reagent Kit V2 Red (Cat#323900, ACD) were purchased from Advanced Cell Diagnostics (ACD, CA, USA). ISH of *circUCK2(2,3)* was performed according to the manufacturer’s instructions. The fluorescent signals (Fast Red) were photo-imaged under a Zeiss Axioplan microscope.

**EdU staining and EdU flow cytometry**

For EdU staining, cells were treated with EdU (10 μM) for 2 hours before fixing, and then stained using the BeyoClick™ EdU-594 (Beyotime, Shanghai, China) according to the manufacture’s protocol. The fluorescent signals were photo-imaged under a Zeiss Axioplan microscope. For EdU flow cytometry, cells which incubated with EdU (10 μM) for 2 h were trypsinized with Trypsin/EDTA (0.25%) and then washed with PBS and permeated with 0.2% Triton-X100. Afterward, cells were incubated with EdU working solution and incorporation was measured by BD LSRII flow cytometer.

**Western Blotting**

Briefly, HCC cells were lysed in RIPA buffer (Epizyme, PC101, China) supplied with protease inhibitor cocktail (Epizyme, GRF101, China) and phosphatase inhibitor (epizyme, GRF102, China) on ice for 20-min. Cell lysates were centrifuged at 12000g for 15-min at 4°C to collect the supernatant. Protein concentrations were determined using BCA assay (Thermo Scientific, 23227, USA). Same amount of protein was separated by SDS-PAGE and transferred onto PVDF membrane. After blocking by 5% BSA in TBST buffer, overnight incubation with primary antibodies, and 2-hour incubation with secondary antibodies, protein bands were detected using Enhanced Pico Light ChemiluminescenceKit (Epizyme, SQ101, China). The information of antibodies used in this study was listed in Supplementary Table 5.

**Cell culture, transfection and infection**

HCC cell lines, PLC/PRF/5, Hep3B, SK-HEP-1, SNU182, SNU398, and SNU449 were purchased from BeinaBio (Beijing, China). Short tandem repeat (STR) profiling was used to validate the identity of cell line (Microread, Beijing, China). Mycoplasma contamination was excluded via a PCR-based method. PLC/PRF/5, Hep3B, and SK-HEP-1 were cultured in Dulbecco's Modified Eagle Medium (Gibco, USA). SNU182, SNU398, and SNU449 cells were cultured in RPMI-1640 medium (Gibco, USA). All the cell culture medium was supplied with 10% fetal bovine serum (FBS) (Gibco, USA), 100 U/ml of penicillin and streptomycin (Invitrogen, USA), 1% non-essential amino acids (Gibco, USA) and 1mM sodium pyruvate (Gibco, USA). Cells were transfection with vectors by using Lipofectamine 2000 (Invitrogen, USA) according to the instruction of manufacturer. siRNAs, miRNA mimics and miRNA inhibitors were transfected into cells with Lipofectamine RNAiMAX (Invitrogen, USA) according to the manufacturer’s instruction. Pseudo lentivirus was prepared by transfecting VSV-G, PAX2, and corresponding lentiviral vectors into 293T cells as previously described. The functional titer of lentivirus was determined according to the protocol previously described. HCC cells were infected with indicated lentivirus with a MOI of 10. Stably infected HCC cells were selected in medium with puromycin for at least one month.

**Polysome fractionation**

For sucrose density gradient preparation, sucrose gradient was made by filling half of centrifuge tube (Beckman) with 5% followed by 50% sucrose buffers from the button. The sucrose mix was applied to the top of a linear 5 to 50% sucrose gradient using a Gradient Master (model 108, BioComp Instruments). PLC/PRF/5 cells were pre-treated with cycloheximide (100μg/ml) for 10 min and lysed by hypotonic lysis buffer containing 0.2U/μl RNase inhibitor, 1x protease inhibitor cocktail (EDTA-free) and 100μg/ml cycloheximide. Cell lysates were loaded on the top of sucrose gradient and were centrifuged using an ultracentrifuge with SW 32 Ti Swinging-Bucket Rotor (Beckman) at 174900g overnight at 4℃. After ultracentrifuge, samples were collected at 750μl/fraction and absorbance was recorded at an OD of 254nm. RNA of each fraction was extracted with TRIzol-LS (Thermo Fisher Scientific) following the manufacturer’s instruction. *CircUCK2(2,3)* levels at each fraction were analyzed by RT-PCR.

**Dual luciferase reporter assay**

To determine the direct interaction between miR-149-5p and *circUCK2(2,3)*, fragments of WT or mutated *circUCK2(2,3)* (carrying mutated miR-149-5p binding site) were cloned into pmirGLO vector (Promega). To determine miR-149-5p target genes, the 3’UTRs of *CNIH4* or *SMAGP* were cloned into pmirGLO (Promega). Luciferase assays were performed in 293T cells. Briefly, 293T cells were seeded at 24-well plates and transfected with 20pmole of miR-149-5p mimics and 200 ng indicated vectors using Lipofectamine^TM^ 2000 (Thermo Scientific, 11668019) according to the manufacturer’s instructions. Luciferase activity was measured 24-hour after transfection using Dual-Luciferase Assay Kit (Promega, E1910) in accordance with the manufacturer’s instructions. To detect the transcriptionally regulatory elements of *circUCK2(2,3)*, the DNA fragments of different lengths upstream of *circUCK2(2,3)* were cloned into pGL3-basic vector (Promega). These constructed vectors (900 ng/well) together with pRL vector as internal control (90 ng/well) were transfected into 293T cells using Lipofectamine^TM^ 2000 (Thermo Scientific, 11668019). The luciferase activity was measured 24-hour after transfection using Dual-Luciferase Assay Kit.

**Enzyme-Linked Immunosorbent Assay (ELISA)**

To determine the level of secreted TGFα protein, we used the QuantiCyto® Human TGF-alpha ELISA kit (Neobioscience, EHC125a.96, China) according to the manufacturer’s instructions. Briefly, cell culture supernatants (100 μl/well) were added into the ELISA plate and incubated at RT for 90-min. The plates were then washed and incubated with the HRP-conjugated antibody for 60-min. After adding substrate solution for a 15-min incubation at 37°C followed by adding stop buffer, the immunoreactivity was determined by measuring absorbance at 450 nm.

**Cell proliferation assays in the context of inhibitors of EGFR downstream signalings**

SNU398 cells (2000 cells/well) were seeded into 96-well cell culture plates and allowed to attach overnight. Cells were then incubated with a MEK inhibitor (Selumetinib, 1μM, HY-50706, MCE), an ERK inhibitor (ASN007, 1μM, HY-136579, MCE), an AKT inhibitor (Perifosine, 5μM, HY-50906 from MCE), or a STAT3 inhibitor (Stattic, 1μM, HY-13818, MCE) for 5 days. Cell viability was measured by CCK-8 assay kit. The information of those inhibitors used in this study was listed in Supplementary Table 5.

**Cytotoxic assay of EGFR inhibitors**

HCC cells (1x10^4^ cells/well) were seeded into 96-well cell culture plates and allowed to attach overnight. Cells were then incubated with EGFR inhibitors at indicated concentration for 48 h. Cell viability was measured by CCK-8 assay kit. The information of EGFR inhibitors used in this study was listed in Supplementary Table 5.

**sgRNA target site selection and *circUCK2(2,3)* knockout**

sgRNA target sites for CRISPR/Cas9 mediated deletion of the *UCK2* intron 1 *AluJb* and *AluSx* elements, and the intron 3 *AluJb* element were selected with CCTop (crispr.cos.uni-heidelberg.de) (pattern: N20NGG, core length = 12, max. core mismatches = 2, max. total mismatches = 4). The transcription of sgRNAs was mediated by the U6 promoter. Therefore, G is necessary at the 5’ end of the respective sgRNAs. The sequence information of sgRNAs was listed in Supplementary Table 4. Knockout of *circUCK2(2,3)* was performed in PLC/PRF/5 cells, which has high endogenous *circUCK2(2,3)* expression. pL-CRISPR.EFS.tRFP vectors co-expressing Cas9 protein and sgRNAs were transfected PLC/PRF/5 cells. 48-72h after transfection, red single cells were sorted directly into 96-well plates containing complete DMEM medium. After 2-week culture, genome DNA of monoclonal lines was collected for PCR validation and sanger sequencing. Genotyping and Sanger sequencing were used to determine the established clones with heterozygous or homozygous deletion.

**Supplementary Figures and Figure Legends**


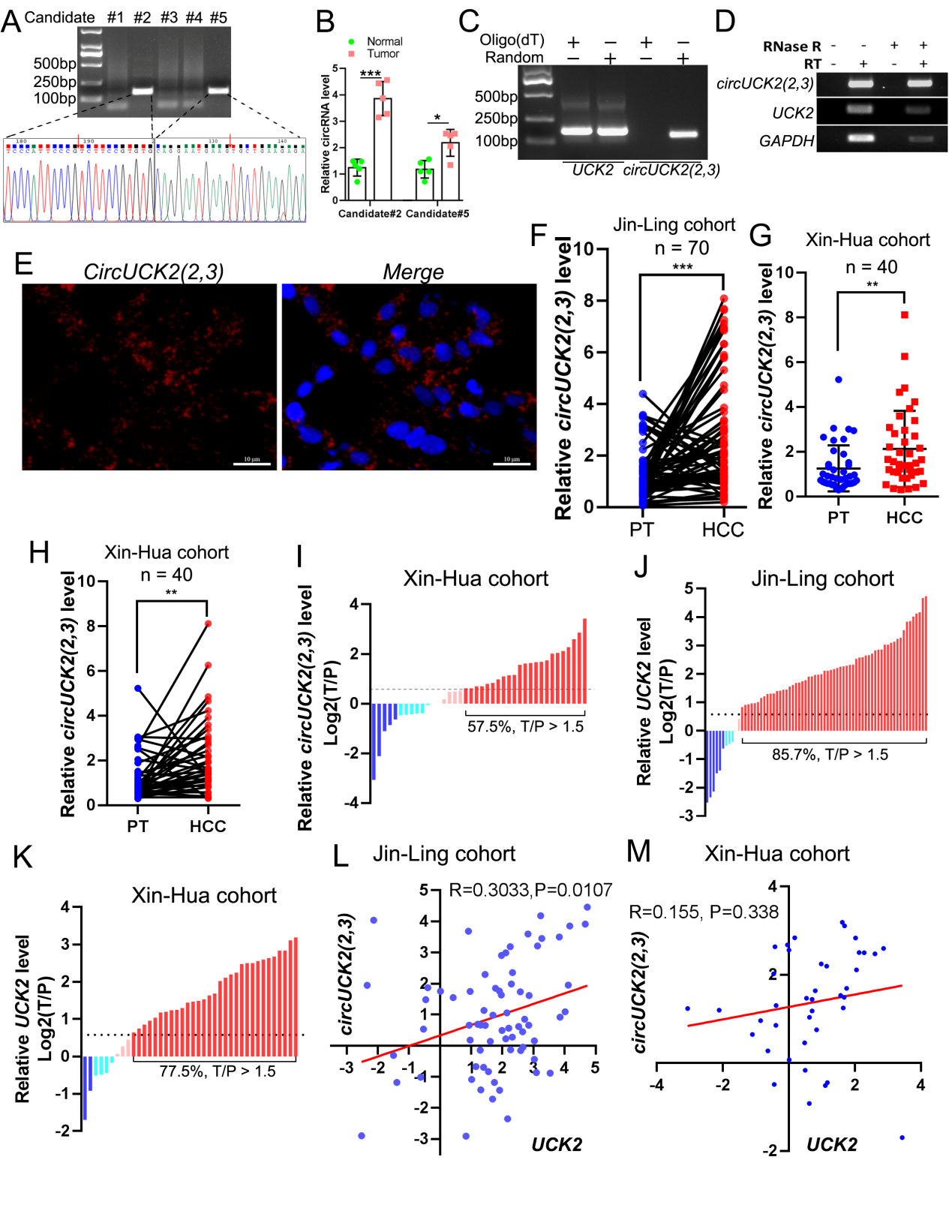


**Figure S1. Identification and characteristics of *circUCK2(2,3)* in HCC tissues**

(A) RT-PCR to validate circRNA candidates selected from circRNA-seq data, and Sanger sequencing results of circRNA candidate#2 and #5 (red arrow indicates the back-splicing site). (B) qRT-PCR to compare expression levels of circRNA candidate#2 and #5 between 5 paired HCC tissues and matched normal tissues. (C) RT-PCR of *circUCK2(2,3)* or *UCK2* by using cDNAs generated from Oligo(dT) or random primers. (D) RT-PCR of *circUCK2(2,3)*, *UCK2*, and *GAPDH* after RNase R treatment. (E) Fluorescent In Situ Hybridization (FISH) of endogenous *circUCK2(2,3)* by using RNAscope assays. (F) Expression levels of *circUCK2(2,3)* in 70 paired HCC and peritumor tissues in the Jin-Ling cohort. (G and H) Expression levels of *circUCK2(2,3)* in 40 paired HCC and peritumor tissues in the Xin-Hua cohort. (I) Tumor vs. peritumor expression ratio of *circUCK2(2,3)* in the Xin-Hua cohort. (J and K) Tumor vs. peritumor expression ratio of *UCK2* in clinical samples of the Jin-Ling cohort (J) and the Xin-Hua cohort (K). (L and M) Expression correlation between *UCK2* and *circUCK2(2,3)* in HCC tissues from the Jin-Ling cohort (L) and the Xin-Hua cohort (M).


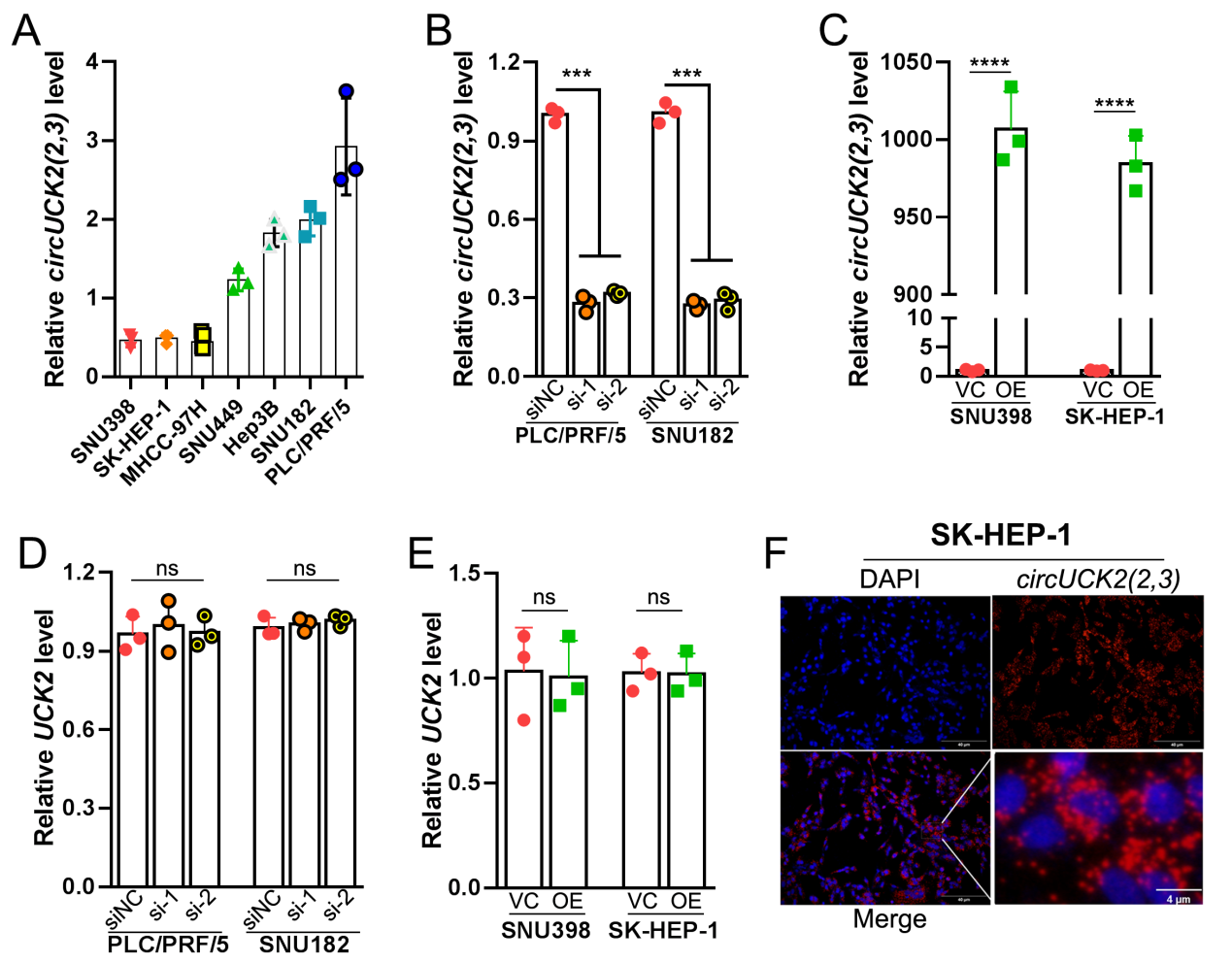


**Figure S2. *CircUCK2(2,3)* knockdown and overexpression**

(A) Expression pattern of endogenous *circUCK2(2,3)* in HCC cell lines. (B and C) qRT-PCR to determine knockdown (B) or overexpression (C) efficiency of *circUCK2(2,3)*. (D and E) qRT-PCR to determine endogenous *UCK2* mRNA levels upon *circUCK2(2,3)* knockdown (D) or overexpression (E). (F) FISH of ectopically expressed *circUCK2(2,3)* by using RNAscope assays in SK-HEP-1 cells.


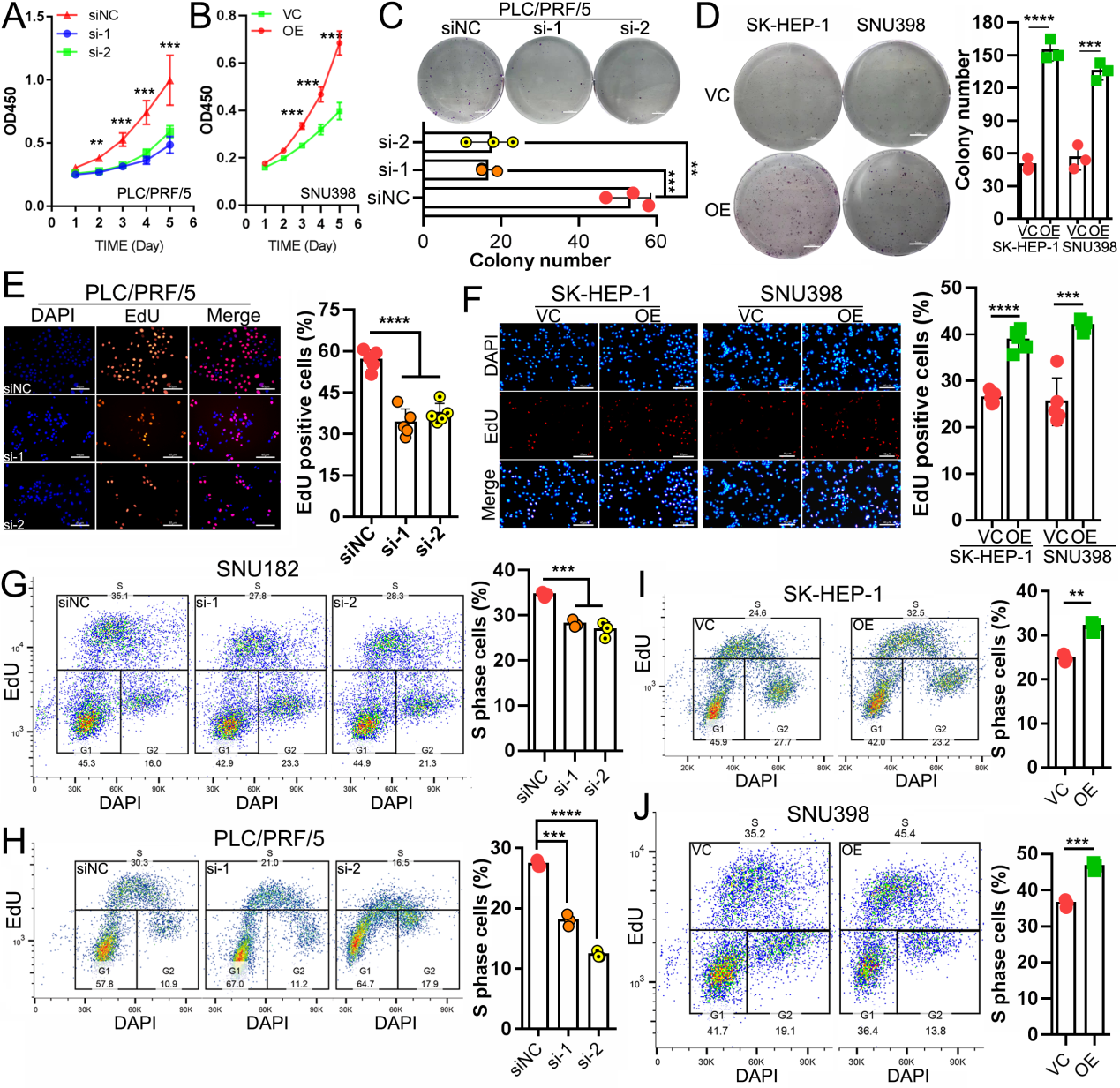


**Figure S3. *CircUCK2(2,3)* promotes HCC cell proliferation**

(A and B) CCK-8 assays in PLC/PRF/5 cells with *circUCK2(2,3)* knockdown (A), or in SNU398 cells with *circUCK2(2,3)* overexpression (B). (C and D) Colony formation assays in PLC/PRF/5 cells with *circUCK2(2,3)* knockdown (C), or in SK-HEP-1 and SNU398 cells with *circUCK2(2,3)* overexpression (D). (E and F) EdU staining in PLC/PRF/5 cells with *circUCK2(2,3)* knockdown (E), or in SK-HEP-1 and SNU398 cells with *circUCK2(2,3)* overexpression (F). (G to J) EdU flow cytometry in SNU182 (G) and PLC/PRF/5 cells (H) with *circUCK2(2,3)* knockdown, or in SK-HEP-1 (I) and SNNU398 cells (J) with *circUCK2(2,3)* overexpression.


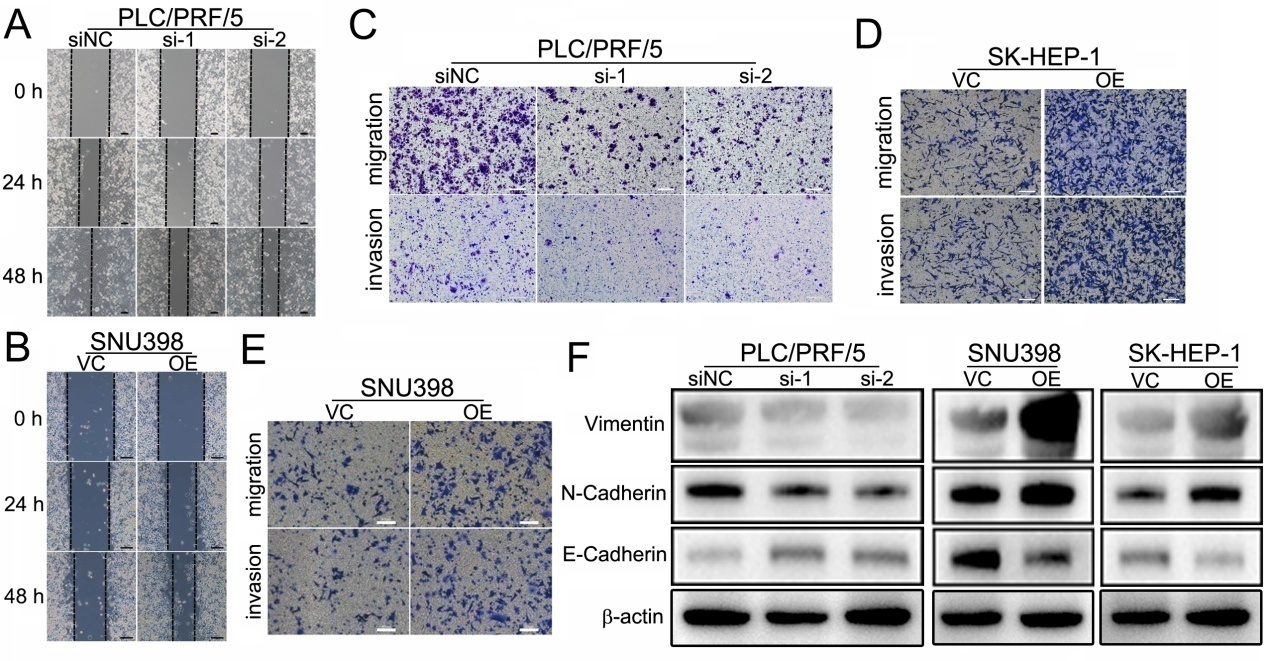


**Figure S4. *CircUCK2(2,3)* promotes HCC cell migration and invasion**

(A and B) Wound healing assays in PLC/PRF/5 cells with *circUCK2(2,3)* knockdown (A) or in SNU398 cells with *circUCK2(2,3)* overexpression (B). (C to E) Transwell migration or invasion assays in PLC/PRF/5 cells with *circUCK2(2,3)* knockdown (C) or in SK-HEP-1 cells (D) or in SNU398 cells (E) with *circUCK2(2,3)* overexpression. (F) Western blotting of EMT markers in PLC/PRF/5 cells with *circUCK2(2,3)* knockdown, or in SNU398 and in SK-HEP-1 cells with *circUCK2(2,3)* overexpression.


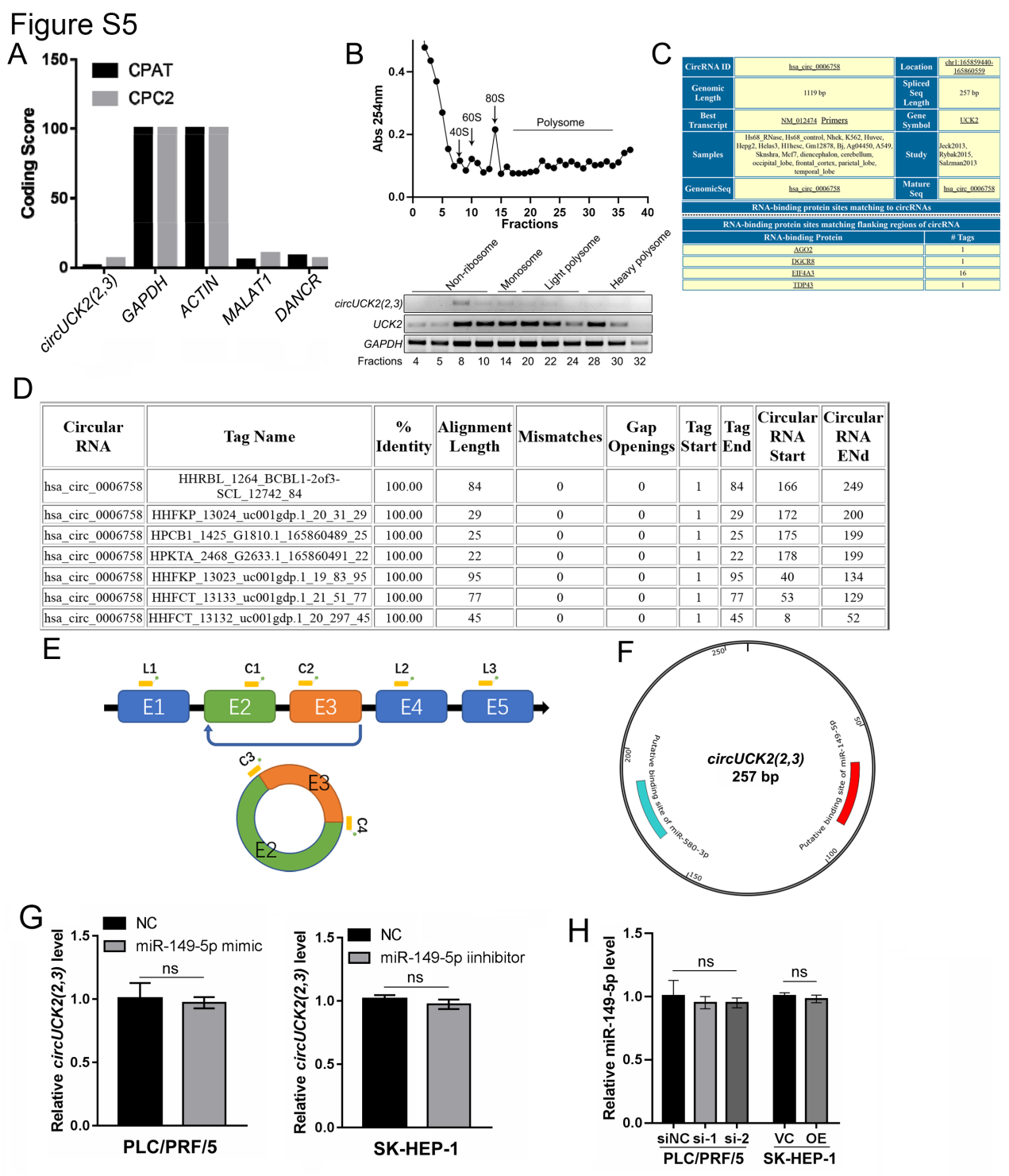


**Figure S5. Bioinformatic analysis of potential functional mechanisms of *circUCK2(2,3)* in HCC**

(A and B) CPAT and CPC2 (A) and polysome fractionation (B) to determine the coding potential of *circUCK2(2,3)*. (C) Using CircInteractome to predict putative interaction proteins of *circUCK2(2,3)*. (D) Six potential AGO2 binding sites on *circUCK2(2,3)*. (E) Schematic showing the locations of probes targeting linear *UCK2* or *circUCK2(2,3)*. (F) Schematic showing the putative binding sites of miR-149-5p and miR-580-3p on *circUCK2(2,3)*. (G) qRT-PCR to detect *circUCK2(2,3)* levels in PLC/PRF/5 cells transfected with miR-149-5p mimics or in SK-HEP-1 cells introduced with miR-149-5p inhibitors. (H) qRT-PCR to detect miR-149-5p levels in PLC/PRF/5 cells with *circUCK2(2,3)* knockdown, or in SK-HEP-1 cells overexpressing *circUCK2(2,3)*.


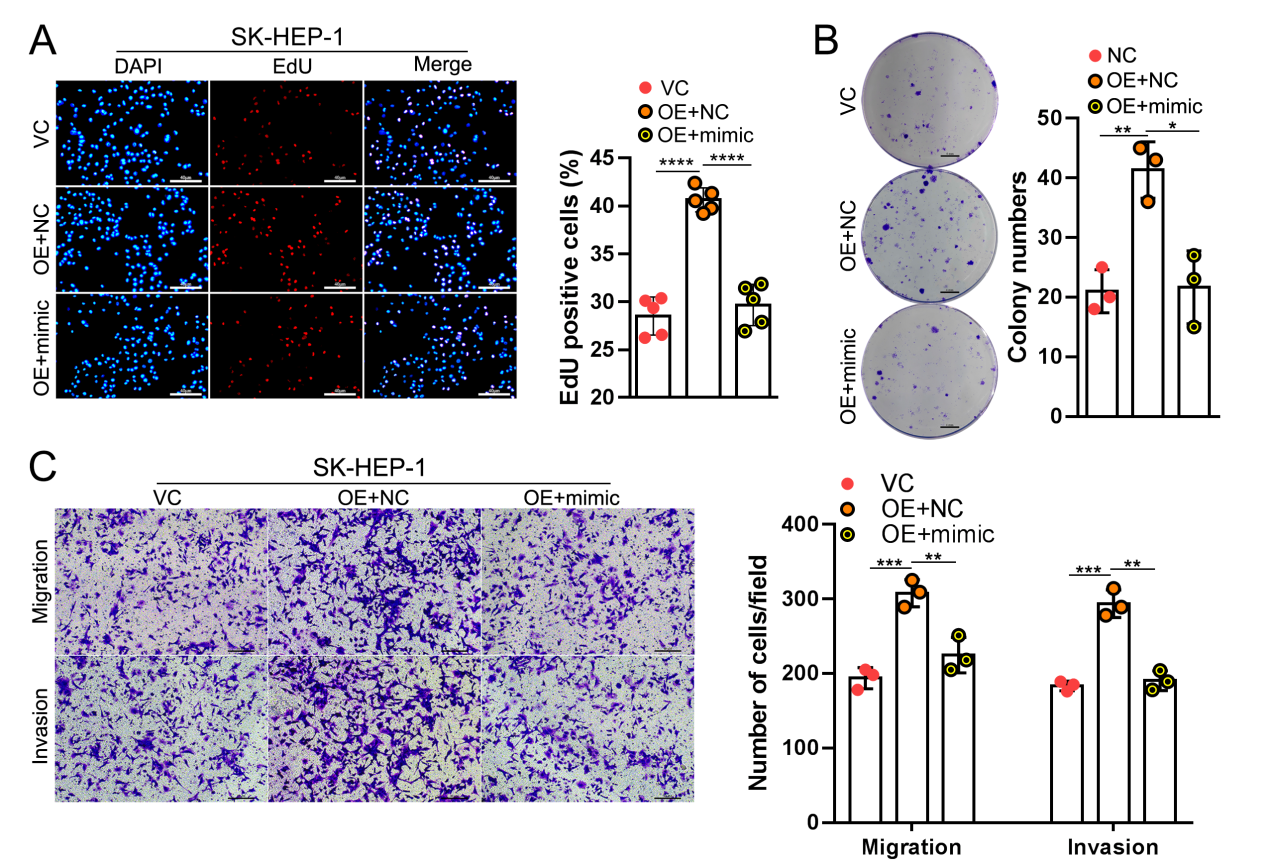


**Figure S6. Introducing miR-149-5p mimics impairs the pro-tumor effect of c*ircUCK2(2,3)* in HCC cells**

(A to C) EdU staining assay (A), colony formation assay (B) and transwell migration/invasion assay (C) in *circUCK2(2,3)* overexpressed SK-HEP-1 cells introduced with negative miRNA controls or miR-149-5p mimics.


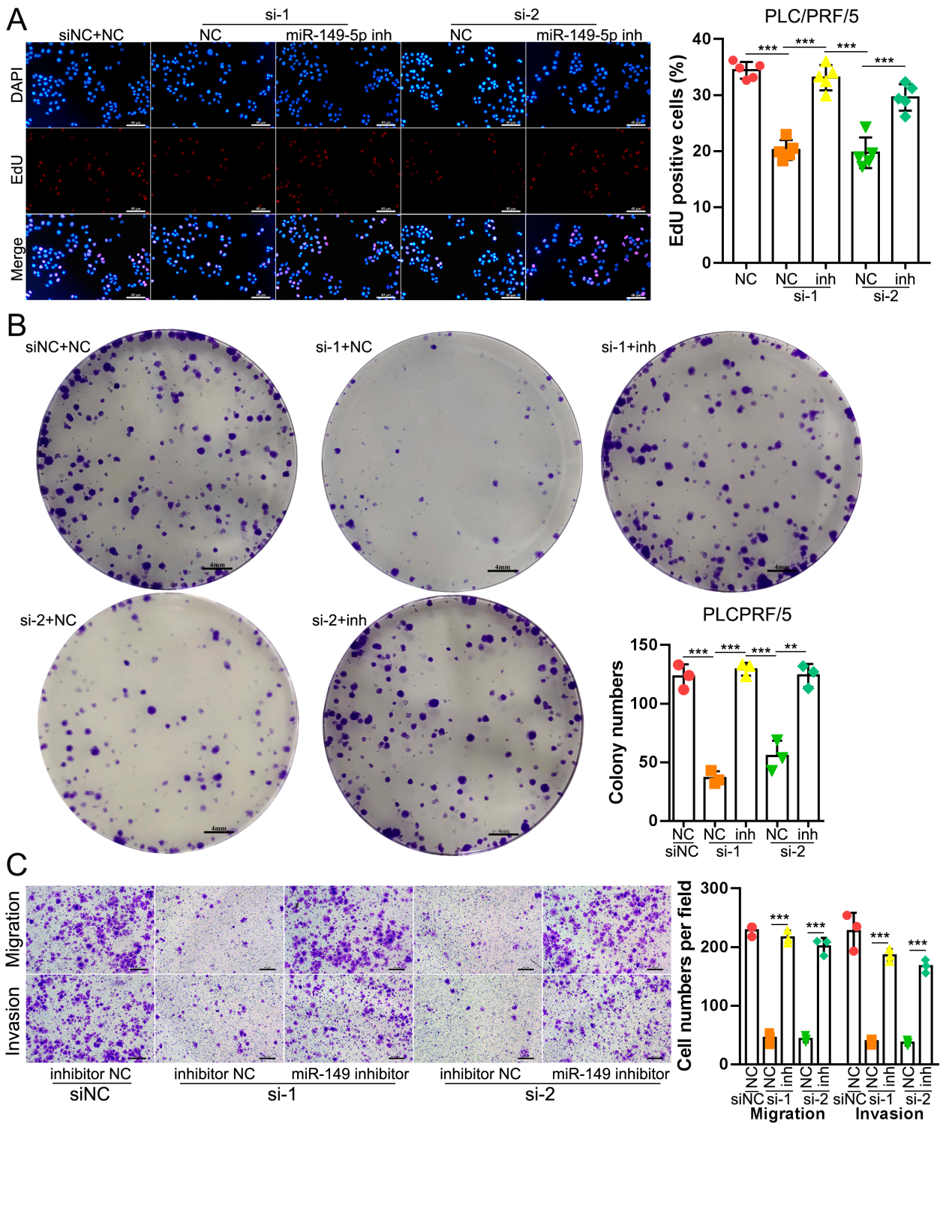


**Figure S7. Introducing miR-149-5p inhibitors rescues HCC cells from *circUCK2(2,3)*-knockdown mediated suppression**

(A to C) EdU staining assay (A), colony formation assay (B), and transwell migration/invasion assay (C) in *circUCK2(2,3)*-knockdown PLC/PRF/5 cells introduced with negative control oligos or miR-149-5p inhibitors.


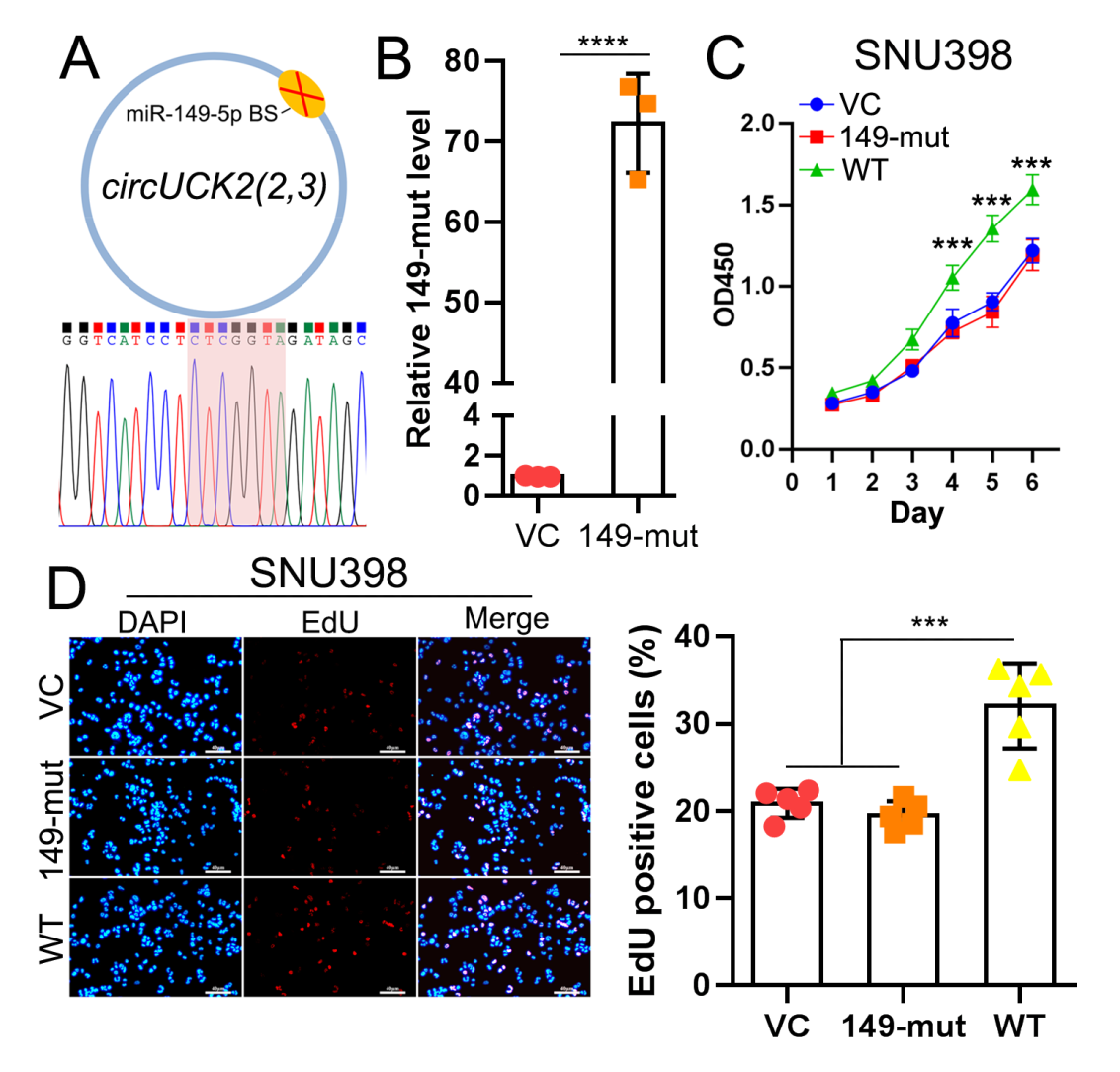


**Figure S8. Sponging miR-149-5p is essential for *circUCK2(2,3)* in promoting HCC cell proliferation**

(A) Schematic and Sanger sequencing showing *circUCK2(2,3)* with mutated miR-149-5p binding site (named 149-mut). (B) qRT-PCR to determine the overexpression of 149-mut. (C and D) CCK8 assays (C) and EdU staining assays (D) in SNU398 cells overexpressing WT or 149-mut *circUCK2(2,3)*.


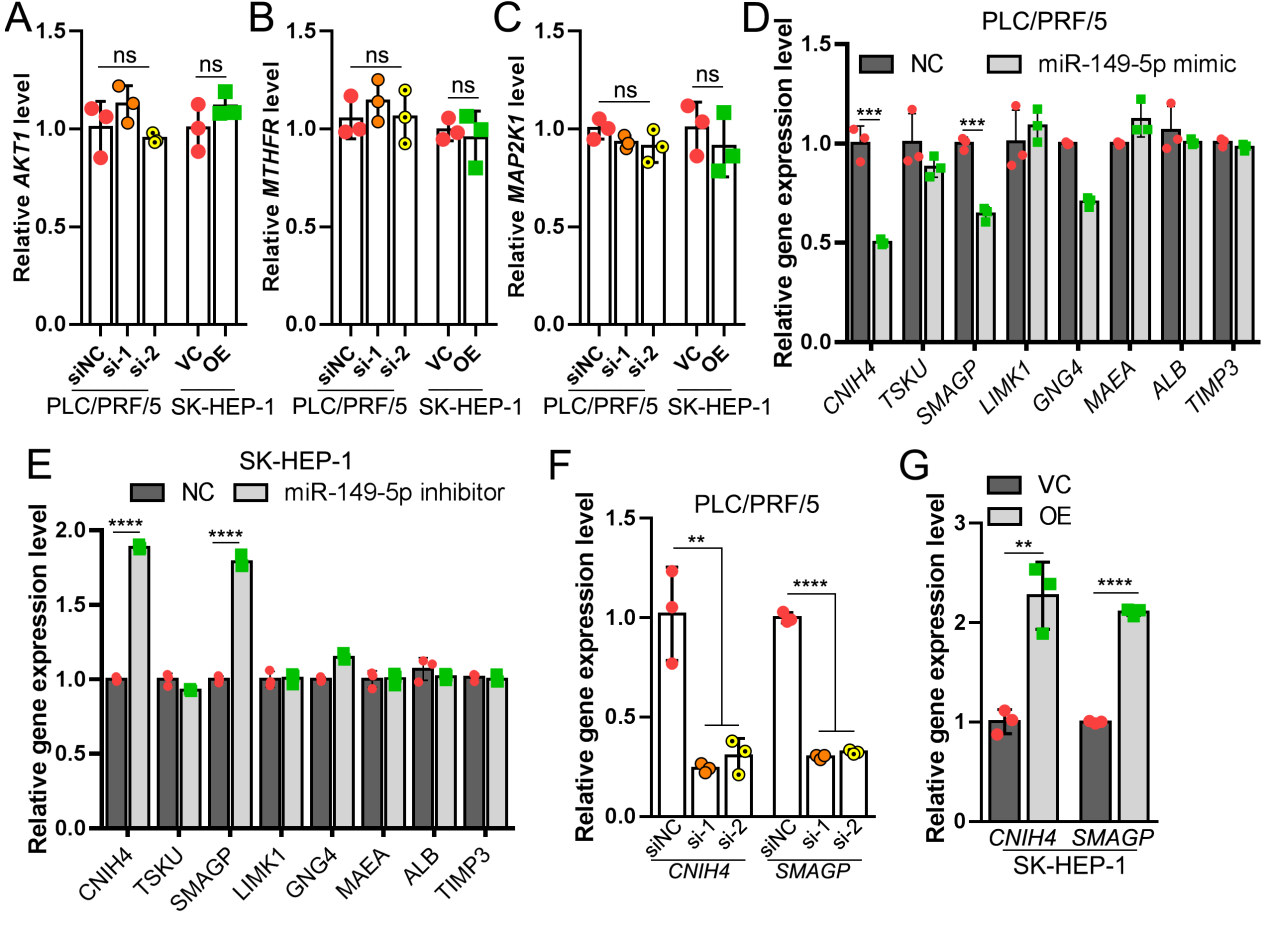


**Figure S9. The potential downstream targets of the *circUCK2(2,3-)*miR-149-5p axis**

(A to C) qRT-PCR to detect expression changes of previously reported miR-149-5p targets, AKT1 (A), MTHFR (B) and MAP2K1 (C). (D and E) qRT-PCR to determine expression changes of indicated genes in PLC/PRF/5 cells introduced with miR-149-5p mimics (D), or in SK-HEP-1 cells transfected with miR-149-5p inhibitors (E). (F and G) qRT-PCR to determine expression changes of indicated genes in PLC/PRF/5 cells with *circUCK2(2,3)* knockdown (F) or in SK-HEP-1 cells with *circUCK2(2,3)* overexpression (G).


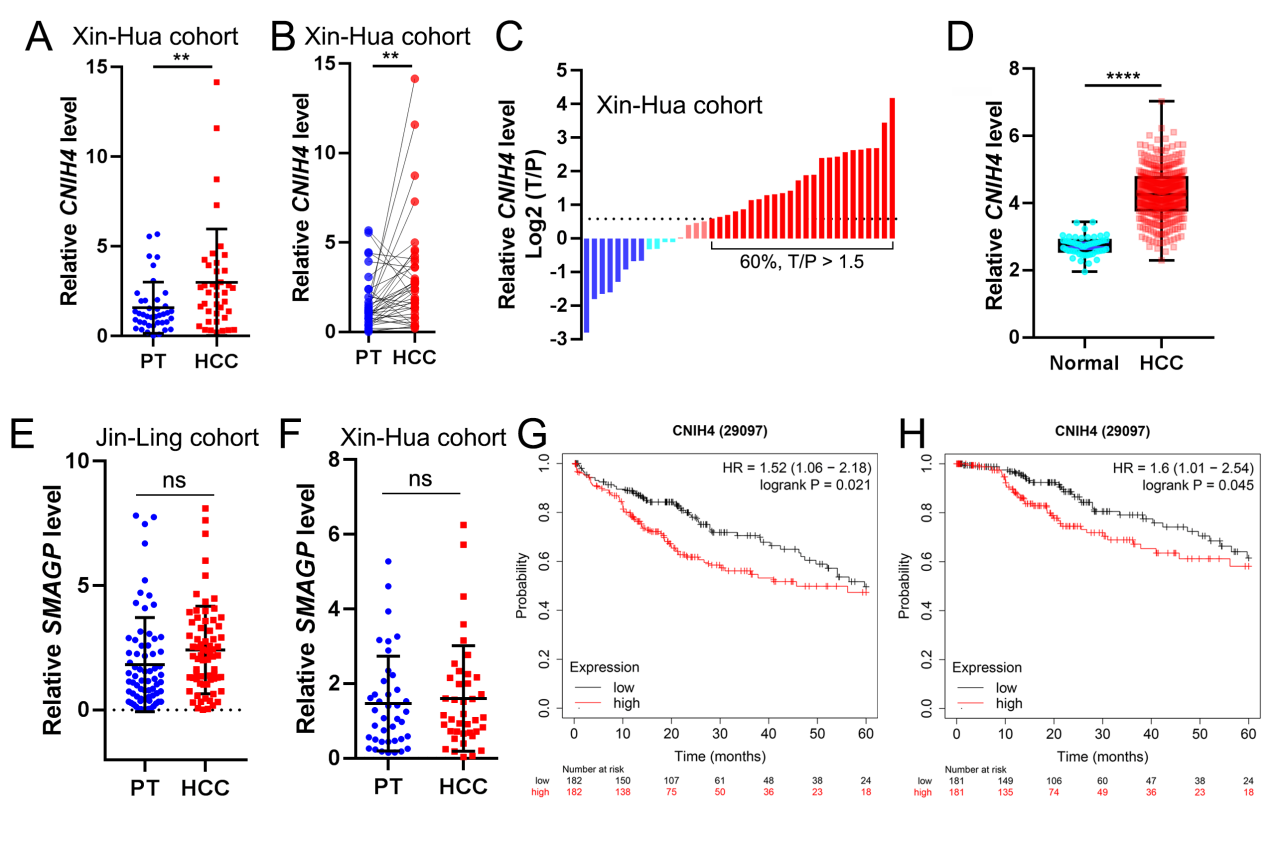


**Figure S10. Expression levels of *CNIH4* and *SMAGP* in HCC samples**

(A and B) Expression levels of *CNIH4* in 40 paired HCC and peritumor tissues in the Xin-Hua cohort. (C) Tumor vs. peritumor expression ratio of *CNIH4* in 40 paired HCC and peritumor tissues in the Xin-Hua cohort. (D) Comparison of *CNIH4* expression levels between normal liver tissues and HCC tissues in the TCGA-LIHC dataset. (E and F) Expression levels of *SMAGP* in the paired HCC and peritumor tissues in the Jin-Ling cohort (E) and the Xin-Hua cohort (F). (G and H) Kaplan-Meier analysis to determine the association of *CNIH4* expression with OS (G) and RFS (H) in HCC patients of the TCGA-LIHC dataset.


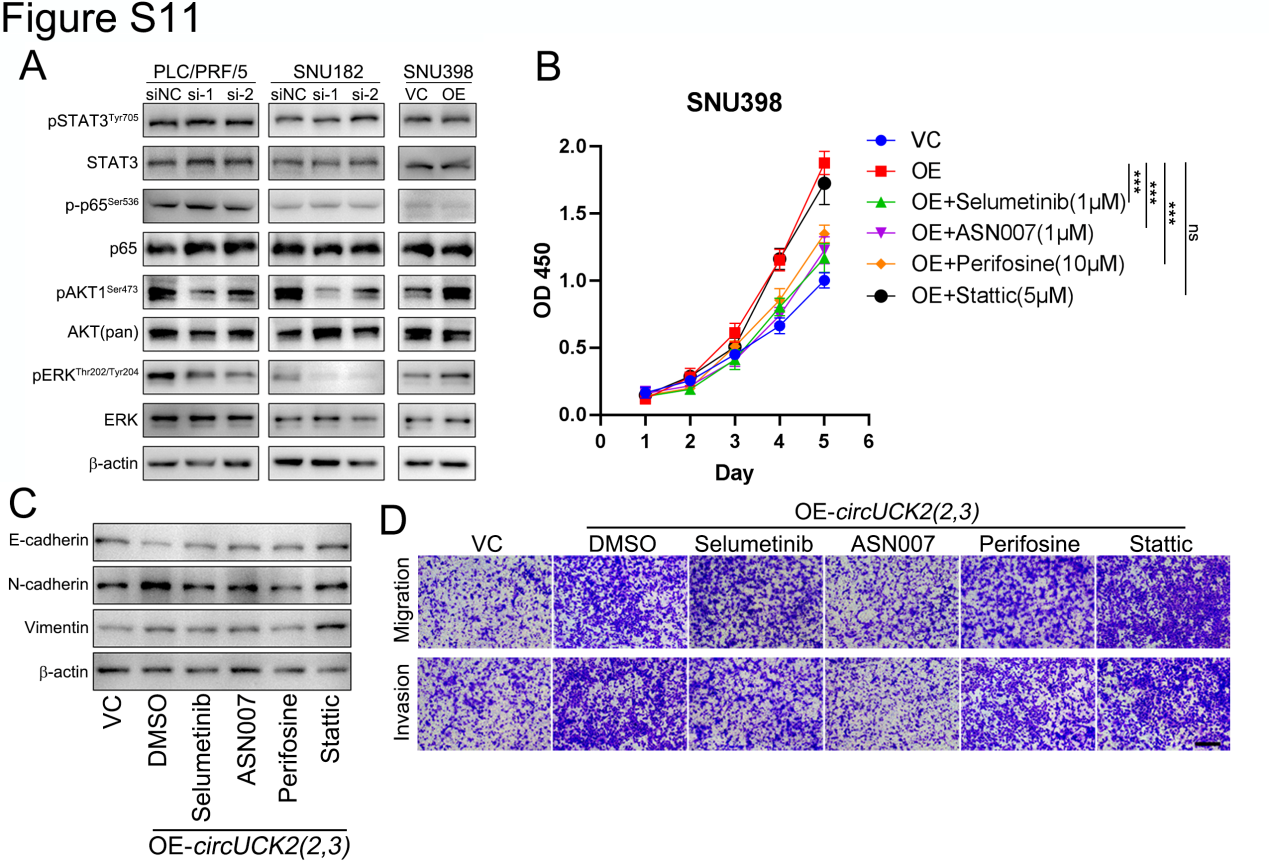


**Figure S11. CircUCK2(2,3) activates EGFR downstream signalings, AKT and ERK.**

(A) Western blotting of EGFR downstream signalings, including STAT3, pSTAT3, p65, p-p65, ERK, pERK, AKT, pAKT, in the context of *circUCK2(2,3)* knockdown or overexpression in HCC cells. (B) CCK-8 assays in SNU398 cells with *circUCK2(2,3)* overexpression treated with a MEK inhibitor (Selumetinib), an ERK inhibitor (ASN007), an AKT inhibitor (Perifosine), and a STAT3 inhibitor (Stattic). (C) Western blotting of EMT markers in SNU398 cells with *circUCK2(2,3)* overexpression treated with indicated inhibitors. (D) Trans-well migration and invasion assays in SNU398 cells with *circUCK2(2,3)* overexpression treated with indicated inhibitors.

**
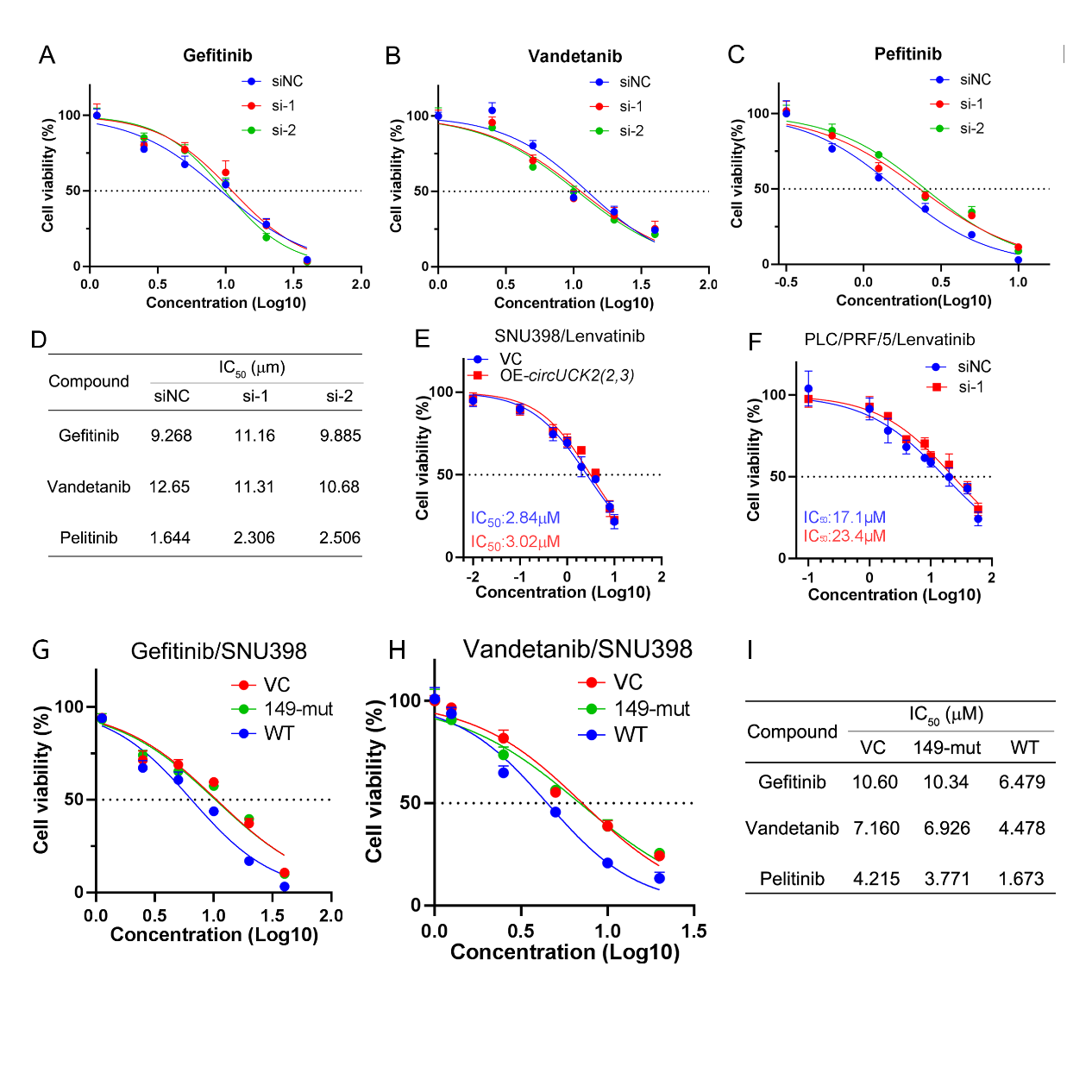
**

**Figure S12. *CircUCK2(2,3)* sensitizes HCC cells to the cytotoxic effects of EGFR inhibitors.**

(A to D) Cytotoxic effects of gefitinib (A), vandetanib (B), and pelitinib (C) in PLC/PRF/5 cells with *circUCK2(2,3)* knockdown. (D) Summary of IC_50_ changes to EGFR inhibitors in PLC/PRF/5 cells with *circUCK2(2,3)* knockdown. (E and F) Cytotoxic effect of lenvatinib in SNU398 cells with or without *circUCK2(2,3)* overexpression (E), or in PLC/PRF/5 cells with or without *circUCK2(2,3)* knockdown (F). (G to I) Cytotoxic effects of gefitinib (G) and vandetanib (H) as well as the summary of IC_50_ changes to EGFR inhibitors in SUN398 cells overexpressing WT or 149-mut *circUCK2(2,3)*.

**
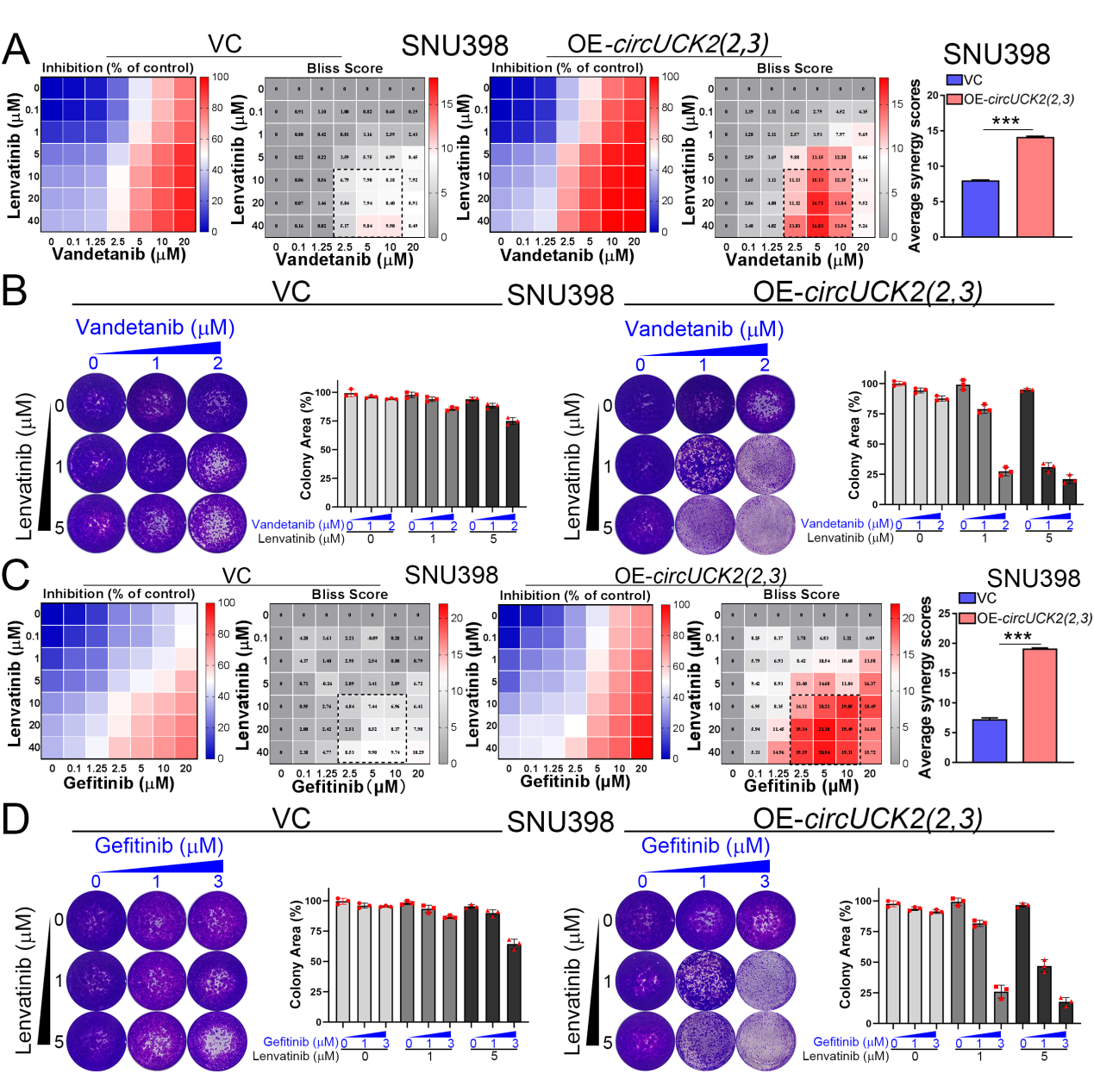
**

**Figure S13. *CircUCK2(2,3)* sensitizes HCC cells to the synergistic killing effects of lenvatinib with EGFR inhibitors**

(A and C) A bliss independent model to evaluate the synergistic killing of lenvatinib with vandatanib (A) and gefitinib (C) in SNU398 cells with or without *circUC*K2(2,3) overexpression. (B and D) The long-term clonogenic synergistic response to the combination of lenvatinib with vandatanib (B) and gefitinib (D) in SNU398 cells with or without *circUC*K2(2,3) overexpression.

**
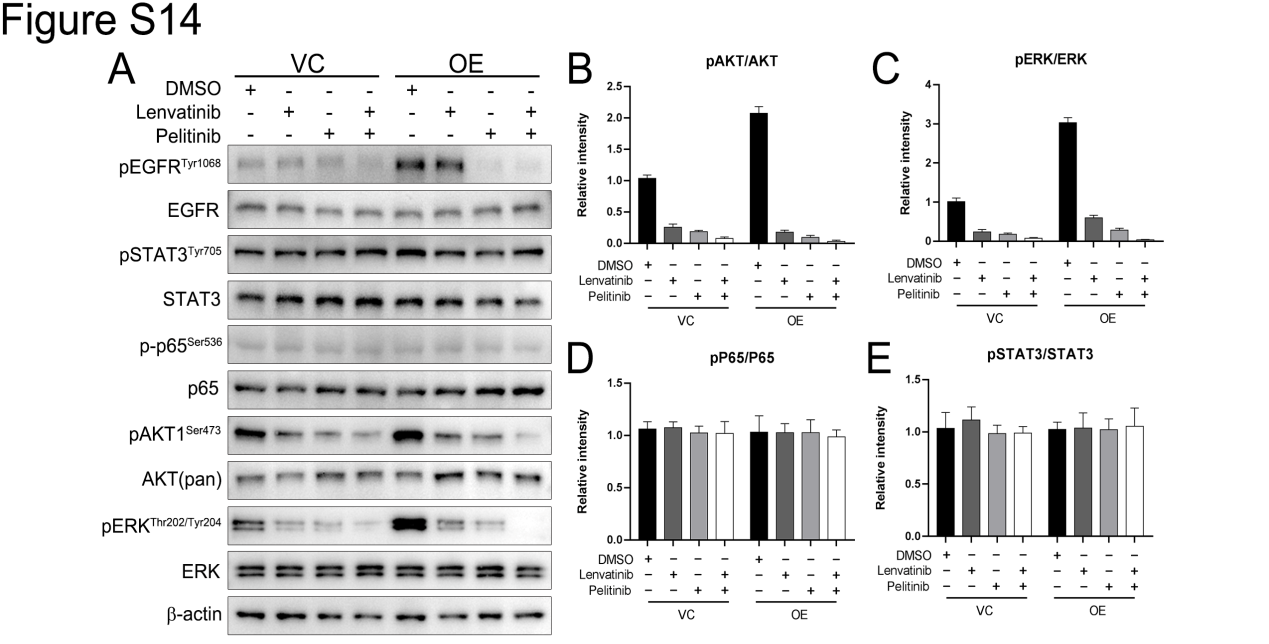
**

**Figure S14. Combined lenvatinib and pelitinib treatment shows greater inhibition on AKT and ERK signaling pathways.**

(A) Western blotting of the activation of EGFR downstream signalings upon lenvatinib, pelitinib, or combined lenvatinib and pelitinib treatment in SNU398 cells. (B to E) Densitometry analyses to illustrate the activation changes of AKT (B), ERK (C), NF-kB (D) and STAT3 (E) pathways upon indicated treatments.

**
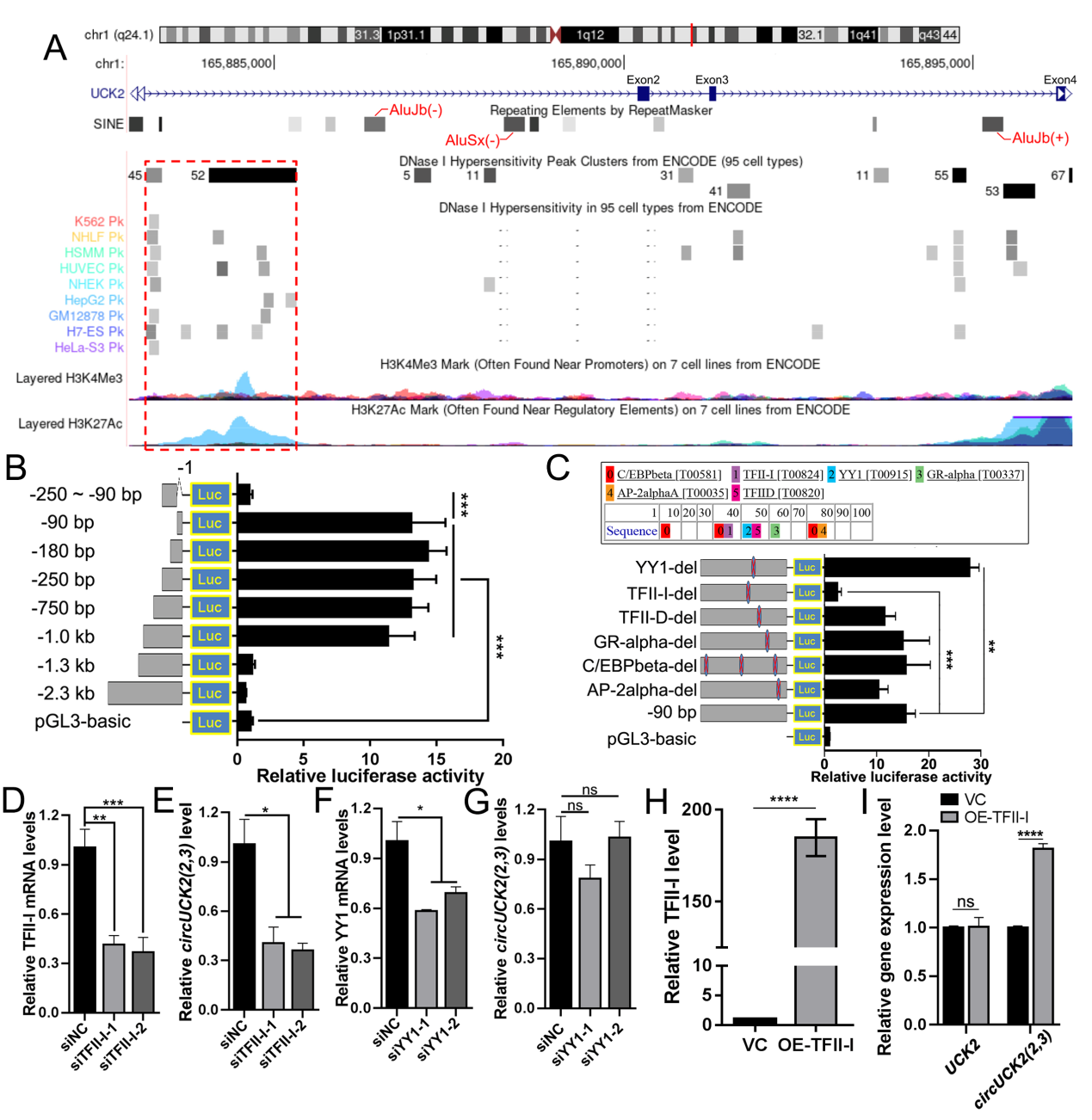
**

**Figure S15. Transcriptional regulatory factors of *circUCK2(2,3)*.**

(A) Genomic localization of putative transcriptional regulatory region of *circUCK2(2,3)* (marked by a red-dashed rectangle) and the flanking *Alu* elements of *circUCK2(2,3)* (labeled in red). (B) The upstream fragments at indicated sizes of *circUCK2(2,3)* were cloned into pGL3-basic vector, and promoter activity of each fragment was determined by luciferase activity assays. (C) The predicted transcriptional factor (TF) binding sites on the core promoter (-1 to -90bp) of *circUCK2(2,3)*. The 90bp core promoter fragments with deletions of indicated TFs were cloned into pGL3-basic vector, and promoter activity was determined by luciferase activity assays. (D and F) qRT-PCR to detect the knockdown efficiency of TFII-I (D) and *YY1* (F). (E and G) qRT-PCR to determine the expression changes of endogenous *circUCK2(2,3)* in response to the knockdown of TFII-I (E) and *YY1* (G). (H) qRT-PCR to determine the overexpression efficiency of TFII-I. (I) qRT-PCR to determine the expression changes of endogenous *UCK2* and *circUCK2(2,3)* in response to TFII-I overexpression.


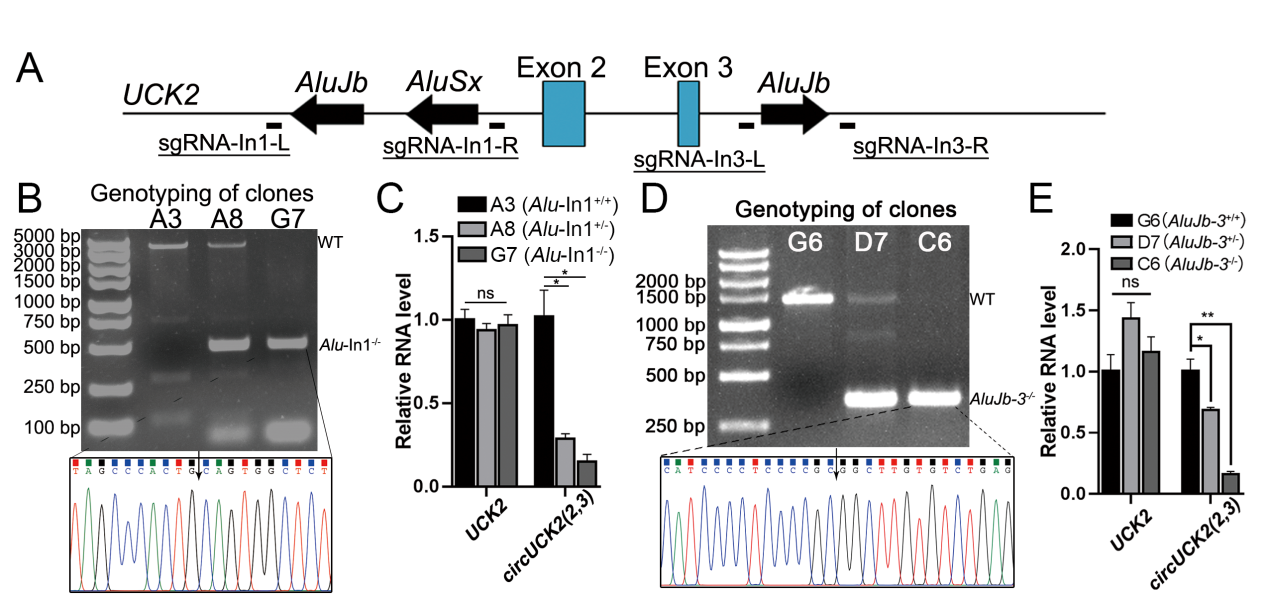


**Figure S16. Flanking ICIs affecting *circUCK2(2,3)* biosynthesis.**

(A) Diagram showing the flanking *Alu* elements of *circUCK2(2,3)* and the location of sgRNAs targeting the indicated *Alu* elements*.* (B) Genotyping of PLC/PRF/5 clones (A3, A8, G7) with heterozygous or homozygous removal of intron 1 *AluJb* and *AluSx*. Sanger sequencing confirmed the removal of intron 1 *AluJb* and *AluSx* (black arrow indicates the junction site)*.* (C) qRT-PCR to detect the expression changes of endogenous *UCK2* and *circUCK2(2,3)* in clones (A3, A8 and G7). (D) Genotyping of PLC/PRF/5 clones (G6, D7, C6) with heterozygous or homozygous deletion of intron 3 *AluJb*. Sanger sequencing confirmed the removal of the intron 3 *AluJb* element (black arrow indicates the junction site). (E) qRT-PCR to detect the expression changes of endogenous *UCK2* and *circUCK2(2,3)* in PLC/PRF/5 clones (G6, D7, C6).
