# Supplementary material for: CircUCK2(2,3) promotes cancer progression and enhances synergistic cytotoxicity of lenvatinib with EGFR inhibitors via activating CNIH4–TGFα–EGFR signaling
Source: Cell Mol Biol Lett. 2025 Jan 30;30:15. doi: 10.1186/s11658-025-00690-1 (PMC11781035; doi:10.1186/s11658-025-00690-1)
Supplement: Supplementary file 2 — Supplementary material 2. [file 11658_2025_690_MOESM2_ESM.docx]

**Supplementary Tables**

**Supplementary Table 1. Correlation between *circUCK2(2,3)* and clinicopathologic characteristics of HCC patients in the Jin-Ling cohort (Related to Figure 1).**

| **特征** | **All cases** | ***CircUCK2*(2,3), T/P < 1.5, n=30** | ***CircUCK2(2,3)*, T/P > 1.5, n=40** | **p value** |
| --- | --- | --- | --- | --- |
| **Gender** |  |  |  | 0.9999 |
| female | 13 | 6 | 7 |  |
| male | 57 | 24 | 33 |  |
| **Age(year)** |  |  |  | **0.0299*** |
| <60 | 32 | 9 | 23 |  |
| ≥60 | 38 | 21 | 17 |  |
| **Serum AFP, ng/mL** |  |  |  | 0.9999 |
| ≤25 | 39 | 17 | 22 |  |
| >25 | 31 | 13 | 18 |  |
| **Serum ALT, U/L** |  |  |  | 0.2455 |
| ≤40 | 56 | 22 | 34 |  |
| >40 | 14 | 8 | 6 |  |
| **Serum AST, U/L** |  |  |  | 0.5359 |
| ≤40 | 57 | 23 | 34 |  |
| >40 | 13 | 7 | 6 |  |
| **Serum TBIL, μM** |  |  |  | 0.7957 |
| ≤17.1 | 50 | 22 | 28 |  |
| >17.1 | 20 | 8 | 12 |  |
| **HBsAg** |  |  |  | **0.0398*** |
| positive | 49 | 25 | 24 |  |
| negative | 21 | 5 | 16 |  |
| **Liver cirrhosis** |  |  |  | 0.3305 |
| yes | 59 | 27 | 32 |  |
| no | 11 | 3 | 8 |  |
| **Tumor size (cm)** |  |  |  | 0.9999 |
| ＜5 | 42 | 18 | 24 |  |
| ≥5 | 28 | 12 | 16 |  |
| **Tumor number** |  |  |  | 0.2828 |
| single | 61 | 28 | 33 |  |
| multiple | 9 | 2 | 7 |  |
| **Encapsulation invasion** |  |  |  | 0.8036 |
| present | 25 | 10 | 15 |  |
| absent | 45 | 20 | 25 |  |
| **Tumor differentiation** |  |  |  | **0.0256*** |
| Ⅰ-Ⅱ | 28 | 17 | 11 |  |
| Ⅲ-Ⅳ | 42 | 13 | 29 |  |

T/P, tumor vs. peritumor; AFP, alpha-fetoprotein; ALT, alanine aminotransferase; AST, aspartate transferase; TBIL, total bilirubin; HBsAg, hepatitis B surface antigen. *P<0.05* was considered statistically significant by Chi-square test.

**Supplementary Table 2. Univariate and multivariate analyses of factors associated with RFS of HCC patients in Jin-Ling cohort (Related to Figure 1).**

| **Variables** | **Categories** | **Univariate analysis** | | | **Multivariate analysis** | | |
| --- | --- | --- | --- | --- | --- | --- | --- |
|  |  | **HR** | **95% CI** | **P value** | **HR** | **95% CI** | **P value** |
| Age | ≤60 vs. ＞60 | 1.174 | 0.5722-2.408 | 0.662 |  |  |  |
| Tumor size | ≤5cm vs. ＞5cm | 1.939 | 0.9189-4.09 | 0.0822***** | 2.771 | 1.26-6.095 | **0.0113*** |
| Tumor differentiation | Ⅰ,Ⅱ vs. Ⅲ,Ⅳ | 0.8686 | 0.3972-1.899 | 0.724 |  |  |  |
| AFP | ≤20ng/ml vs. >20ng/ml | 0.7795 | 0.3763-1.614 | 0.502 |  |  |  |
| HbsAg | positive vs. negative | 0.8856 | 0.4041-1.941 | 0.761 |  |  |  |
| Encapsulation invasion | positive vs. negative | 1.329 | 0.6432-2.745 | 0.443 |  |  |  |
| *CircUCK2(2,3)* | T/P>1.5 vs. T/P<1.5 | 2.201 | 1.006-4.817 | 0.0483***** | 2.969 | 1.295-6.807 | **0.0102*** |

AFP, alpha-fetoprotein; HBsAg, hepatitis B surface antigen; T/P, tumor vs. peritumor. *P<0.1* was considered statistically significant in the univariate analysis for further multivariate analysis, *P<0.05* was considered statistically significant in the multivariate analysis.

**Supplementary Table 3. Sequence information of PCR primers and cloning oligos**

|  | Gene | Direction | Sequence (5’ to 3’) |
| --- | --- | --- | --- |
| Primers for RT-PCR and qRT-PCR | *CircUCK2(2,3)* | Forward | CAGGATAGCTTCTACCGTGTCC |
|  |  | Reverse | GATAGTCCACCTCATTCTGCCC |
|  | *circSMARCA5* | Forward | CAAGATGGGCGAAAGTTCA |
|  |  | Reverse | GCACCTCTTTCCAAAATACCA |
|  | *UCK2* | Forward | AGCTAGCGGCAAGTCTTC |
|  |  | Reverse | TCTGCTCCGAGGTAAGGACA |
|  | *GAPDH* | Forward | GGAGCGAGATCCCTCCAAAAT |
|  |  | Reverse | GGCTGTTGTCATACTTCTCATGG |
|  | *CNIH4* | Forward | TCAACTTACCTGTTGCCACTTG |
|  |  | Reverse | TCTGTTGGATCAAACACTCCCA |
|  | *SMAGP* | Forward | CACAGCACTCATTGCAGTTGTTA |
|  |  | Reverse | TCATAGGTGACGTAGCTGCC |
|  | *TSKU* | Forward | GAGACCTTCGGCCTTTTCGAC |
|  |  | Reverse | CGCCAACACCGACTCATTC |
|  | *MAEA* | Forward | GCGGCTCAGTTGTCCATGA |
|  |  | Reverse | TTTGTTCAGCGTCTCGTAGGG |
|  | *LIMK1* | Forward | GACGTTCCTCAAGGAGGTGA |
|  |  | Reverse | AGAGCACCCCGATGAACTTG |
|  | *GNG4* | Forward | GAGGGCATGTCTAATAACAGCAC |
|  |  | Reverse | AGACCTTGACCCTGTCCATAC |
|  | *ALB* | Forward | GATGAGATGCCTGCTGACTTGC |
|  |  | Reverse | CACGACAGAGTAATCAGGATGCC |
|  | *TIMP3* | Forward | TACCGAGGCTTCACCAAGATGC |
|  |  | Reverse | CATCTTGCCATCATAGACGCGAC |
|  | *MALAT1* | Forward | AGCAGACACACGTATGCGAA |
|  |  | Reverse | GTGGTTCCCAATCCCCACAT |
|  | *BIRC5* | Forward | AGGACCACCGCATCTCTACAT |
|  |  | Reverse | AAGTCTGGCTCGTTCTCAGTG |
|  | *18S* | Forward | GGAGAGGGAGCCTGAGAAACG |
|  |  | Reverse | TTACAGGGCCTCGAAAGAGTCC |
|  | *AKT1* | Forward | CTGCACAAACGAGGGGAGTA |
|  |  | Reverse | GCGCCACAGAGAAGTTGTTG |
|  | *MTHFR* | Forward | CCGCCGTGAACTACTGTGG |
|  |  | Reverse | AGATGGCCCGTGATCTCCTC |
|  | *MAP2K1* | Forward | CAATGGCGGTGTGGTGTTC |
|  |  | Reverse | GATTGCGGGTTTGATCTCCAG |
|  | *YY1* | Forward | GTTCAGGGATAACTCGGCCA |
|  |  | Reverse | TTCGAACGTGCACTGAAAGG |
|  | TFII-I | Forward | GAAGATGCTGCGAGACCAGT |
|  |  | Reverse | TCACACGACCACCTACATGC |
| Primers for *circUCK2(2,3)* promoter cloning | P-*circUCK2(2,3)* | Reverse | TTTTTTCTCGAGGGTTTGAGAAGGCAGGCAATC |
|  | P-*circUCK2(2,3)(2,3)*-2.3K | Forward | TTTTTTGGTACCAGGGCAAATTACTGGTGC |
|  | P-*circUCK2(2,3)*-1.3K | Forward | TTTTTTGGTACCAGCATGTCTAGTGGACCCTGG |
|  | P-*circUCK2(2,3)*-1K | Forward | TTTTTTGGTACCATCGGGGTGAATGCCAAAG |
|  | P-*circUCK2(2,3)*-750bp | Forward | TTTTTTGGTACCTTTAACCCTCCCGACTCC |
|  | P-*circUCK2(2,3)*-500bp | Forward | TTTTTTGGTACCGTTTGCCATCAGTGGTGTGG |
|  | P-*circUCK2(2,3)*-250bp | Forward | TTTTTTGGTACCATGACCAAATTCCAGAACCC |
|  | P-*circUCK2(2,3)*-180bp | Forward | TTTTTTGGTACCACTTTCCGTTTCTATGAATTTGAC |
|  | P-*circUCK2(2,3)*-90bp | Forward | TTTTTTGGTACCTATAATTGTTAAGTCTTAGCCCACTG |
|  | P-*circUCK2(2,3)*- 90bp-del | Reverse | TTTTTTGGTACCTTAATAGTCACAAAAAGACAAATACC |
|  | C/EBPbeta-del | Forward | TTTTTTGGTACCTATAATAAGTCTTAGCCCACTGTAACAGACCCTTTCCCATTTTAGATTTCTATAGGCTC |
|  |  | Reverse | TTTTTTCTCGAGGGTTTGAGAAGGCAGTCCTGAGCCTATAGAAATCTAAAATGGGAAAGGG |
|  | GR-alpha-del | Forward | TTTTTTGGTACCTATAATTGTTAAGTCTTAGCCCACTGTAACAGTTGTACCCTTTCCCATTTTAGATTTCTCTCAGGATTG |
|  |  | Reverse | TTTTTTCTCGAGGGTTTGAGAAGGCAGGCAATCCTGAGAGAAATCTAAAATGGGAAAGGGTAC |
|  | TFIID-del | Forward | TTTTTTGGTACCTATAATTGTTAAGTCTTAGCCCACTGTAACAGTTGTACCCTTTCCCATTTCTATAGGCTC |
|  |  | Reverse | TTTTTTCTCGAGGGTTTGAGAAGGCAGGCAATCCTGAGCCTATAGAAATGGGAAAGGGTAC |
|  | TFII-I-del | Forward | TTTTTTGGTACCTATAATTGTTAAGTCTTAGCCCACTGTAACAGTTGTACCCATTTTAGATTTCTATAGGCTC |
|  |  | Reverse | TTTTTTCTCGAGGGTTTGAGAAGGCAGGCAATCCTGAGCCTATAGAAATCTAAAATGGGTACAACTG |
|  | YY1-del | Forward | TTTTTTGGTACCTATAATTGTTAAGTCTTAGCCCACTGTAACAGTTGTACCCTTTCTTTTAGATTTCTATAGGCTC |
|  |  | Reverse | TTTTTTCTCGAGGGTTTGAGAAGGCAGGCAATCCTGAGCCTATAGAAATCTAAAAGAAAGGGTAC |
|  | AP-2alpha-del | Forward | TTTTTTGGTACCTATAATTGTTAAGTCTTAGCCCACTGTAACAGTTGTACCCTTTCCCATTTTAGATTTCTATAGGCTC |
|  |  | Reverse | TTTTTTCTCGAGGGTTTGAGAAGAATCCTGAGCCTATAGAAATCTAAAATGG |
| Primers for constructing luciferase reporter vectors | *CNIH4* | Forward | ACGAGCTCGCTAGCCTCGAGAGCTGGAGAAGCCGTGGTTG |
|  |  | Reverse | CCTGCAGGTCGACTCTAGAACATACAGAGGATGAAAAAC |
|  | *SMAGP* | Forward | ACGAGCTCGCTAGCCTCGAGCCAGTGCCATCGTCCAGATG |
|  |  | Reverse | CCTGCAGGTCGACTCTAGAAGGTTGAAGGGAGAGAAAAG |
|  | *circUCK2(2,3)* | Forward | ACGAGCTCGCTAGCCTCGAGTCTTCCGTGTGTGCTAAGAT |
|  |  | Reverse | CCTGCAGGTCGACTCTAGACGGGAATGGGAGACAAAGTC |
| Primers for *TFII-I* expression vector | TFII-I | Forward | TTTGCTAGCATGGACTACAAAGACGATGACGACAAGGCCCAAGTTGCAATGTCCAC |
|  |  | Reverse | TTTGCGGCCGCTACCACGTGGGGTCTGGTTC |
| Oligos for sgRNA plasmids | sgRNA-In1-L | Forward | CACCgAAAGGGTACAACTGTTACAG |
|  |  | Reverse | AAACCTGTAACAGTTGTACCCTTTC |
|  | sgRNA-In1-R | Forward | CACCGGTAGCCCTACCTTTATCAG |
|  |  | Reverse | AAACCTGATAAAGGTAGGGCTACC |
|  | sgRNA-In3-L | Forward | CACCgAAACTCAGACACAAGCCTGT |
|  |  | Reverse | AAACACAGGCTTGTGTCTGAGTTTC |
|  | sgRNA- In3-R | Forward | CACCgCAATTGCCTCAGGACTGGCG |
|  |  | Reverse | AAACCGCCAGTCCTGAGGCAATTGC |
| Primers for genotyping | *Alu*-In1-del | Forward | TGAGCCCAGTACCTAGCATA |
|  |  | Reverse | CCCCAAAGTTCCTTCCTCCC |
|  | *AluJb*-In3-del | Forward | TTTTTCCCGTCTTCCCTGCC |
|  |  | Reverse | CACCAAAGAGCCAAGGGTGA |

**Supplementary Table 4. Sequence information of siRNAs, shRNAs, ACD probes, *circUCK2(2,3)* and linear *UCK2* pull-down probes and sgRNAs**

| siRNA negative control (siNC in paper) | UUCUCCGAACGUGUCACGUTT |
| --- | --- |
| si-*circUCK2(2,3)*-1 (si-1 in paper) | CCAUUCCCGUCUUCCGUGUTT |
| si-*circUCK2(2,3)*-2 (si-2 in paper) | UCCCGUCUUCCGUGUGUGCTT |
| si-TFII-I-1 | CGAGAACUAUGAUCUUGCATT |
| si-TFII-I-2 | GUCGUGUGAUGGUAACAGATT |
| si*YY1*-1 | GCCUCUCCUUUGUAUAUUATT |
| si*YY1*-2 | CCCAAACAACUGGCAGAAUTT |
| si-*CNIH4* | GCUGAAGUCACACAUGAAAGATT |
| *ACD-Z-UCK2* | TCCCATTCCCGTCTTCCGTGT |
| *ACD-Z-PPIB* | TGTAATCAAGGACTTCATGATCC |
| *ACD-Z-DaPI* | GGAEPMLESDVLKKWQQODI |
| *UCK2*-L1 | TAGACAGTAACTGTCTCCTC |
| *UCK2*-L2 | CAGATTATCTGCACCTCTAG |
| *UCK2*-L3 | CAGGAATCTGTACTTCCAGA |
| *UCK2*-C1 | CGATCTTAGCACACACGGAA |
| *UCK2*-C2 | CGGGTGGTCAAAGTTGAACT |
| *UCK2*-C3 | AAGTCATACACGGGGATCTG |
| *UCK2*-C4 | ACGGAAGACGGGAATGGGAG |

**Supplementary Table 5. Antibodies used in this study**

| **Name** | **Company** | **Catalog #** |
| --- | --- | --- |
| Phospho-EGF Receptor (Tyr1068) (D7A5) XP® Rabbit mAb | Cell Signaling Technology | #3777 |
| EGF Receptor (D38B1) XP® Rabbit mAb | Cell Signaling Technology | #4267 |
| E-Cadherin (24E10) Rabbit mAb | Cell Signaling Technology | #3195 |
| N-Cadherin (D4R1H) XP® Rabbit mAb | Cell Signaling Technology | #13116 |
| Vimentin (D21H3) XP® Rabbit mAb | Cell Signaling Technology | #5741 |
| Rabbit (DA1E) mAb IgG XP® Isotype control | Cell Signaling Technology | #3900 |
| Argonaute 2 (C34C6) Rabbit mAb | Cell Signaling Technology | #2897 |
| ERK | Proteintech | 83533-1-RR |
| pERK(Thr202/Tyr204) | Proteintech | 28733-1-AP |
| STAT3 | Proteintech | 10253-2-AP |
| pSTAT3(Tyr705) | Cell Signaling Technology | 9145 |
| P65 | Cell Signaling Technology | 8242 |
| p-p65(Ser536) | Cell Signaling Technology | 3033 |
| AKT (pan) | Cell Signaling Technology | 4691 |
| pAKT (Ser473) | Cell Signaling Technology | 4060 |
| β-Actin Mouse Monoclonal Antibody | Beyotime | AF0003 |
| Ki67 Rabbit Monoclonal Antibody | Beyotime | AF1738 |
| HRP-labeled Goat Anti-Rabbit IgG(H+L) | Beyotime | A0208 |
| HRP-labeled Goat Anti-Mouse IgG(H+L) | Beyotime | A0216 |
| Polyclonal Anti-CNIH4 Antibody | ATLAS ANTIBODIES | HPA044268 |
| Lenvatinib | Selleckchem | S1164 |
| Gefitinib | Selleckchem | S1025 |
| Vandetanib | MCE | HY-10200 |
| Pelitinib | Selleckchem | S1392 |
| Selumetinib | MCE | HY-50706 |
| ASN007 | MCE | HY-136579 |
| Perifosine | MCE | HY-50906 |
| Stattic | MCE | HY-13818 |
